# Challenges of using and managing medication: a meta-ethnography of the experiences and perceptions of people with intellectual disability and people who support them

**DOI:** 10.1136/bmjopen-2024-090876

**Published:** 2025-09-18

**Authors:** Iman Ghosh, Danielle Adams, Peter Auguste, Anna Brown, Eddie Chaplin, Samantha Flynn, Julia Gauly, Paramjit Gill, Peter E Langdon, David Mahon, Kerry Martin, Stephen Patterson, Daniel Sutherland, Samuel J Tromans, Yen-Fu Chen, Kate Seers, Supporting Medication use In people with a LEarning disability (SMILE), Eddie Chaplin

**Affiliations:** 1 Centre for Evidence and Implementation Science, University of Birmingham, Birmingham, UK; 2 Warwick Applied Heath, University of Warwick, Coventry, UK; 3 Centre for Research in Intellectual and Developmental Disabilities (CIDD), University of Warwick, Coventry, UK; 4 London South Bank University Institute of Health and Social Care, London, UK; 5 Foundation for People with Learning Disabilities, London South Bank University, London, UK; 6 Intellectual Disabilities Research Institute (IDRIS), University of Birmingham, Birmingham, UK; 7 Birmingham Community Healthcare NHS Foundation Trust, Birmingham, UK; 8 Herefordshire and Worcestershire Health and Care NHS Trust, Worcester, UK; 9 People First Dorset, Dorset, UK; 10 Sunderland People First, Sunderland, UK; 11 SAPPHIRE Group, Department of Population Health Sciences, University of Leicester, Leicester, UK; 12 Adult Learning Disability Service, Leicestershire Partnership NHS Trust, Leicester, UK; 13 Department of Health and Welfare, University of Taipei, Taipei, Taiwan

**Keywords:** QUALITATIVE RESEARCH, Delivery of Health Care, Integrated, Disabled Persons, Medication Reconciliation, Medication Adherence, Person-Centered Care

## Abstract

**Abstract:**

**Objective:**

To explore the challenges experienced by people with intellectual disability, their carers and health and social care professionals when using and managing medication.

**Design:**

A synthesis of qualitative research using meta-ethnography.

**Data source:**

We searched seven databases: MEDLINE, Embase, CINAHL, Science, Social Science and Conference Proceedings Citation Indices (Web of Science), Cochrane Library, PsycINFO and Proquest Dissertations and Theses from inception to September 2022 (updated in July 2023).

**Eligibility criteria for selecting studies:**

We included studies exploring the challenges and perceptions of people with intellectual disability, their carers and health and social care professionals regarding medication management and use.

**Results:**

We reviewed 7593 abstracts and 475 full texts, resulting in 45 included papers. Four major themes were identified: (1) Medication-related issues, (2) navigating autonomy and relationships, (3) knowledge and training needs and (4) inequalities in the healthcare system. We formulated a conceptual framework centred around people with intellectual disability and described the interconnectedness between them, their carers and health and social care professionals in the process of managing and using medication. We identified challenges that could be associated with the person, the medication and/or the context, along with a lack of understanding of these challenges and a lack of capability or resources to tackle them. We developed an overarching concept of ‘collective collaboration’ as a potential solution to prevent or mitigate problems related to medication use in people with intellectual disability.

**Conclusions:**

The effective management of medication for people with intellectual disability requires a collaborative and holistic approach. By fostering person-centred care and shared decision-making, providing educational and practical support, and nurturing strong relationships between all partners involved to form a collective collaboration surrounding people with intellectual disability, improved medication adherence and optimised therapeutic outcomes can be achieved.

**PROSPERO registration number:**

CRD42022362903.

**STRENGTHS AND LIMITATIONS OF THIS STUDY**

- This is the first evidence synthesis of qualitative research related to medication use for people with intellectual disability using meta-ethnography, an approach that is particularly suitable for exploring experiences and perceptions.
- We conducted comprehensive searches covering seven databases and used GRADE-CERQual (Grading of Recommendations Assessment, Development and Evaluation working group - Confidence in Evidence from Reviews of Qualitative research) to assess the trustworthiness of our findings.
- We present our findings as conceptual frameworks and a line of argument that are easy to understand by a wide range of audiences.
- Our synthesis was undertaken mainly through the lens of UK healthcare and we may have missed some studies given the broad scope of the topic.
- The synthesis was restricted by available literature, which was dominated by research from high-income, western countries and lacked diversity in relation to culture, ethnicity, level of disability and settings.

## Introduction

 Intellectual disability (also known as learning disability in the UK) is described as ‘a group of aetiologically diverse conditions originating during the developmental period characterised by significantly below-average intellectual functioning and adaptive behaviour that are approximately two or more standard deviations below the mean, based on appropriately normed, individually administered standardised tests’.^1^ The global prevalence of intellectual disability is around 1%, but is twice that within low- and middle-income countries relative to high-income nations.^2^ The recent Global Burden of Disease Study found that the age-standardised rate of idiopathic intellectual disability alone (ie, excluding intellectual disability associated with conditions such as fetal alcohol syndrome and Down syndrome) in 2021 was 1157.2 per 100,000 people, with a slightly higher occurrence among males compared with females.^3^ People with intellectual disability often have a heightened likelihood of being autistic, compared with the general population.^4^ Moreover, they frequently experience multiple physical and mental health conditions, leading to a lifespan that is up to 23 years shorter than those without intellectual disability.^5^ It is therefore unsurprising that this group is often prescribed multiple medications,^6 7^ including psychotropic medications, leading to higher levels of polypharmacy relative to the general population.^8^ This could lead to overmedication, increasing the risk of adverse effects such as obesity, constipation and visual impairment.^8 9^ Furthermore, people with intellectual disability may experience discrimination, and healthcare professionals sometimes fail to recognise and diagnose health conditions among this group of people due to diagnostic overshadowing—failure to recognise an underlying physical condition (such as cancer and cardiovascular disease) or a mental illness because of misattribution of its symptoms (or a person’s reaction to the symptoms) to the person’s intellectual disability or related behaviours, leading to delayed diagnosis and a lack of effective treatment for treatable conditions and further increase in health inequality.^10^,^15^ Medication management for people with intellectual disability is a complex process, involving multidisciplinary teams based in diverse health and social care settings. Early diagnosis and prompt treatment are essential for improving prognosis in various physical and mental health conditions commonly seen in this group.^16^ People with intellectual disability often require substantial support in taking their medications. They also can face challenges in understanding their medications, including their purpose, method of administration and timing, which frequently results in poor adherence. Besides adherence, caregivers and practitioners may have concerns about medication side effects and adequate monitoring.^17^

Addressing these multifaceted issues related to medicine management and optimisation is an important research agenda for improving the well-being and reducing health inequalities experienced by people with intellectual disability. Interviews with healthcare professionals in care homes have highlighted challenges and conflicts in providing support, while a systematic review on pain management suggests pain is often undertreated despite widespread polypharmacy.^18 19^ A few other reviews have also contributed valuable insights, although they tended to focus narrowly on specific conditions or medications and have adopted a narrative approach to summarising findings from previous studies without an attempt to conduct higher-level synthesis. For instance, previous reviews have explored the management of diabetes; understanding of psychotropic medication; and the training needs of mainstream healthcare professionals. However, they frequently fail to consider the perspectives of support workers or the broader context of care.^20^,^25^

Meta-ethnography is a rigorous approach to developing meaningful interpretations from a collection of qualitative studies.^26^ To our knowledge, no existing review has applied this method specifically to explore medication-related issues within this population. We used this approach to explore the perceptions and experiences of the use and management of medication among people with intellectual disability, their family and paid carers, and health and social care professionals. Through examination of qualitative evidence from different groups of people involved in the use and management of medications for people with intellectual disability across different conditions and indications using meta-ethnography, this review aimed to develop an in-depth and more holistic understanding of potential challenges in optimising medication use among people with intellectual disability and to suggest ways to overcome the challenges, which could provide new insights that might not have been uncovered in previous reviews adopting narrower scopes and narrative synthesis approaches.

## Materials and method

We applied Noblit and Hare’s (1988) seven stage approach to meta-ethnography which includes the following steps: getting started, deciding what is relevant, reading the studies, determining how studies are related, translating studies into each other, synthesising translations and expressing the synthesis.^27^ This approach has many strengths as described by Soundy and Heneghan (2022), including bringing together experiential data from multiple studies, identifying factors influencing experience and perceptions in relation to phenomena of interest, enhancing understanding of participants’ perspectives, helping understand why interventions may not work in practice despite research evidence of efficacy, and generation of theories.^28^ The seven steps used were:

### Stage 1: getting started

To facilitate medicines optimisation, it is essential to understand the challenges faced by those with intellectual disability throughout the pathway of medication use and the interpersonal dynamics between them and the people who support or care for them. Our research team collectively had a good understanding of qualitative evidence synthesis methods and experience in working with people with intellectual disability. Further, we consulted with two patient and stakeholder groups (described below) and a Project Advisory Group who provided valuable input at all stages of the meta-ethnography. The Project Advisory Group consisted of an adult with intellectual disability, representatives from charities in the UK that support people with intellectual disability and healthcare professionals, including a consultant clinical psychologist, a consultant intellectual disability psychiatrist, a general practitioner, a nurse and a pharmacist who is also a family carer. This review was conducted as a part of a larger mixed-method evidence synthesis and the protocol was pre-registered with PROSPERO (CRD42022362903).^29^

### Stage 2: deciding what is relevant

To create an effective search for qualitative studies, we designed our eligibility criteria to focus on the challenges associated with medication use and management, with a specific emphasis on understanding the perceptions and experiences of people with intellectual disability and the people who support or care for them. The full eligibility criteria are reported in table 1. An information specialist (AB) conducted a comprehensive literature search across multiple electronic databases: MEDLINE All (via Ovid), Embase (Ovid), CINAHL (EbscoHost), Science, Social Science and Conference Proceedings Citation Indices (Web of Science), Cochrane Library (all databases, via Wiley), PsycINFO (Ovid) and ProQuest Dissertations and Theses Global. This search encompassed records from the inception of those databases up to 19 September 2022, and it was subsequently updated on 11 July 2023. We checked the reference list of all the eligible systematic reviews for relevant publications and performed citation tracking for other relevant studies. The MEDLINE search strategy is provided in online supplemental material 1.

**Table 1:** Eligibility criteria for the meta-ethnography

Sample	Children, adolescents and adults with intellectual disabilities, their carers and health and social care professionals who support them
Phenomenon of interest	People with intellectual disability and a physical long-term condition (eg, diabetes, chronic obstructive pulmonary disease, arthritis, hypertension), for which there is currently no cure, and which are managed with drugs or other treatment. or People with intellectual disability and mental health issues and/or challenging behaviour for which medication is prescribed.
Design	Qualitative studies or mixed methods research that has a clearly identified and reported qualitative element. Mixed-method studies are included only if it separately reported the qualitative findings.
Evaluation	Outcomes for people with intellectual disability: Experiences and perceptions regarding the optimal use and management of medications (eg, adhering to medication for long-term conditions, mental health issues and challenging behaviour, and of underprescribing and overprescribing of medications, knowledge and understanding of prescribed medication). Outcomes for carers and health and social care professionals: Experiences and perceptions of supporting people with intellectual disability to optimise their use and management of medications (eg, adhering to their medication for chronic diseases, mental health issues and challenging behaviour; dealing with overprescribing or underprescribing of medication and issues related to supply, storage and administration; and improving knowledge and understanding of prescribed medication). Studies reporting relevant qualitative data on these outcomes were considered, whether or not the data were the central focus of the studies (eg, studies focusing on disease management for which medication management is an important part would also be included).
Research type	Studies published in English (or with available English translation). Studies from low- and middle-income countries would be included when considered applicable to the UK, in particular, when the healthcare system is comparable to the UK.
Notes on search terms used	We used comprehensive terms related to intellectual disability in our search strategy. Language changes over time and varies geographically and by community. There are many cases where antiquated, non-standard, exclusionary and potentially offensive terms for intellectual disability have been used in past and present literature. In light of this, we included such terms in the search strategy presented in online supplemental material 1 in order to conduct a sensitive, comprehensive search for relevant studies, while recognising and acknowledging the inappropriate and harmful nature of these terms.^85^

Title and abstract screening involved two independent reviewers from a pool of five (IG, DS, DA, JG, NA) using predefined inclusion and exclusion criteria (table 1) in Covidence.^30^ Potentially relevant full texts underwent double screening by two independent reviewers from a pool of four (IG, DS, DA, NA), and any discrepancies were addressed through discussion or by engaging a third reviewer (Y-FC or KS). The Critical Appraisal Skills Programme (CASP) Checklist for Qualitative Studies was used by one reviewer (IG) to critically appraise all included papers, and a second independent reviewer (DA) critically appraised a random sample of 10% of included papers to check the accuracy of the appraisals.^31^ Disagreement was resolved through reviewer discussion. To gauge the overall quality of evidence, we calculated the proportion of the studies meeting each CASP item. We used the four domains of the GRADE-CERQual framework (Grading of Recommendations Assessment, Development and Evaluation working group - Confidence in Evidence from Reviews of Qualitative research) including (1) ‘Methodological limitation’; (2) ‘Relevance’; (3) ‘Adequacy of data’ and (4) ‘Coherence’, to appraise reviewers’ confidence in the research findings (the thematic groups generated by our synthesis) and finally to rate the overall confidence as very low, low, moderate and high.^32^ As there is no universally agreed-upon rule for determining the confidence assessment,^33^ we operationalised the assessment of individual GRADE-CERQual domains as follows: the number of relevant studies fulfilling CASP criteria indicated *methodological limitation*; the proportion of studies contributing to the theme out of 45 represented *coherence*; the number of codes supporting each theme suggested the level of *adequacy*; and overall subjective assessment of richness of codes for *relevance*. A theme was considered to have ‘high confidence’ when it demonstrated ‘low or minor concerns’ across all four domains. Conversely, a ‘low confidence’ rating was assigned if there were two or fewer instances of ‘low or minor concerns’ across the four domains (≤2/4).

### Stages 3 and 4: reading the studies and determining how they are related

One reviewer (IG) thoroughly reviewed all the papers and extracted information on population(s), study methodology and the themes with the supporting quotes, using a predefined data extraction form. A second reviewer (DA) conducted duplicate extraction for 10% of the data. Any disagreements were resolved through discussions between the reviewers. A single reviewer (IG) generated and defined codes from a batch of five studies by using NVivo V.12 and then applied across all included studies,^34^ while maintaining an open mind for any new codes that emerged. The codes were then grouped into conceptual categories. IG, DA, Y-FC and KS discussed the codes and conceptual categories in a series of meetings to reach an agreement. A preliminary conceptual model was created to illustrate the relationship between different conceptual categories from the perspectives of different actors involved in the medication use process.

### Stage 5: translating studies into each other

Through comparing and discussing the conceptual categories, four reviewers (IG, DA, Y-FC and KS) were involved in determining the higher-level thematic groups. Once agreed, we reviewed the preliminary conceptual model and thematic groups with the Project Advisory Group and patient and public involvement (PPI) groups to match and finalise them to be included in the line of argument.

### Stages 6 and 7: synthesising translations and expressing the synthesis

Patterns and associations among the initial conceptual categories and thematic groups were then further explored to identify key, cross-cutting aspects of medication use, which were summarised in the second conceptual model. Based on these conceptual models derived from reviewed qualitative evidence and the understanding of the medication use process that we gained from the literature as well as experiences shared by our Project Advisory and PPI groups, we then translated all the key concepts into the line of argument and the overarching concept shown in our final conceptual model. Additionally, we structured our report according to the new Meta-Ethnography Reporting Guidelines (eMERGe) to enhance reporting quality.^35^

### Patient and public involvement

Two PPI groups, supported by a lead (DM), were consulted throughout the duration of this project (see online supplemental material 2). The first PPI group comprised people with intellectual disabilities and the second Stakeholder PPI group comprised family members and health and social care professionals who work with or care for people with intellectual disabilities. They contributed by defining terminologies, sharing their personal experiences, offering insights on emerging findings, and providing feedback on research scope and manuscripts. Additionally, their involvement extended to creating an easy-read version of key findings for wider dissemination purposes.

## Results

The electronic database searches identified 24 818 records. A total of 7593 titles and abstracts were screened after removal of duplicates using EndNote V.21 and Covidence (see online supplemental material figure 1).^30 36^ Following title and abstract screening, 475 studies underwent full text review, among which 44 primary studies and 4 systematic reviews met the eligibility criteria. Three out of the four systematic reviews reported medication use and disease management among people with intellectual disability who had diabetes,^22 24 25^ while the remaining review assessed training needs for healthcare professionals (HCPs) who supported people with intellectual disability.^23^ Analysis and synthesis of data from the primary studies informed our meta-ethnography. We only used the systematic reviews to verify our analysis and no new themes or additional primary studies were identified through the reviewal of the systematic reviews. In addition, we identify an additional eligible study from citation tracking. Overall, 45 studies were included in our meta-ethnography, 42 of which were peer-reviewed journal articles and 3 were doctoral theses.^37^,^39^

The characteristics of the included qualitative studies are summarised in online supplemental material table 1. The studies were conducted across nine different countries. The highest number of articles (n=16) was from the UK,^1738^,^52^ followed by seven from the Netherlands,^53^,^59^ six from Australia,^60^,^65^ five from Ireland,^66^,^70^ four from the USA,^3771^,^73^ three from New Zealand^74^,^76^ and one each from Belgium,^77^ Canada^78^ and Finland.^19^ A single study recruited participants from both the UK and Ireland.^79^ The studies were published between 2005 and 2023 and used a number of qualitative methods, such as semi-structured interviews, focus groups, online questionnaire and analyses of free-text responses from questionnaires and personal diaries. Psychotropic medicines prescribed for challenging behaviour and mental health conditions were most frequently studied (n=16).^1738 39 41^,^45 47 51 56^ Other studies reported experiences and issues related to prescribed medications for diabetes (n=4),^40 54 55 74^ asthma (n=2)^61 62^ and neurological disorders such as epilepsy (n=3).^46 64 79^

The included studies covered perceptions from a wide range of participants such as people with intellectual disabilities (n=16), people who support or care for them (n=28) and HCPs (n=14). About half of the studies (n=22) presented some of the demographic details for people with intellectual disabilities.^4147^,^51 53 55 56 59 61 62 65^ Various levels of intellectual disabilities, such as borderline to mild,^59^ mild,^56 61^ mild-to-moderate^41 48 50 51 54 55 74 76 78^ and severe-to-profound^66^,^68^ were described in the studies. The proportion of females with intellectual disabilities, where reported, ranged from 14% (one female out of seven participants)^59^ to 71%.^50^ Although views from adolescents and adults were well covered, only three studies reported perspectives on behalf of children.^66^,^68^ Only 6^4749^,^51 56 78^ out of the 45 studies reported information on the ethnicity of participants with intellectual disabilities. White ethnicity stood out with the highest proportion in three of these studies,^49 51 78^ while two of the studies included exclusively white population with intellectual disabilities.^50 56^

Across studies, paid and unpaid carers, including family members, and others such as service managers, direct support professionals and support workers were described using different terms. For brevity, we refer to them collectively as ‘carers’.

We applied the CASP qualitative research checklist to critically appraise the quality of the included studies and incorporated the findings to inform the ‘methodological limitation’ domain of the GRADE-CERQual (online supplemental material table 2). Overall, the quality of the studies was good. 6 of the 45 included studies^17 38 60 62 65 76^ fulfilled 9/10 CASP criteria, while 14^19 39 40 45 51 57 61 63 67 69 71 72 75 77^ fulfilled 8/10. Only five studies^37 42 47 53 78^ met 5/10 or less of the CASP criteria. Findings from these five studies were consistent with other studies, although they did not provide any additional themes. It was noticed that 27/45 studies did not clearly justify the appropriateness of the research design to address the aims of the research, and only 1 study was judged to have adequately considered the relationship between researcher and participants.^38^ A table summarising the quality appraisal results is included in online supplemental material table 3.

### Synthesis of findings

**Figure 1:**
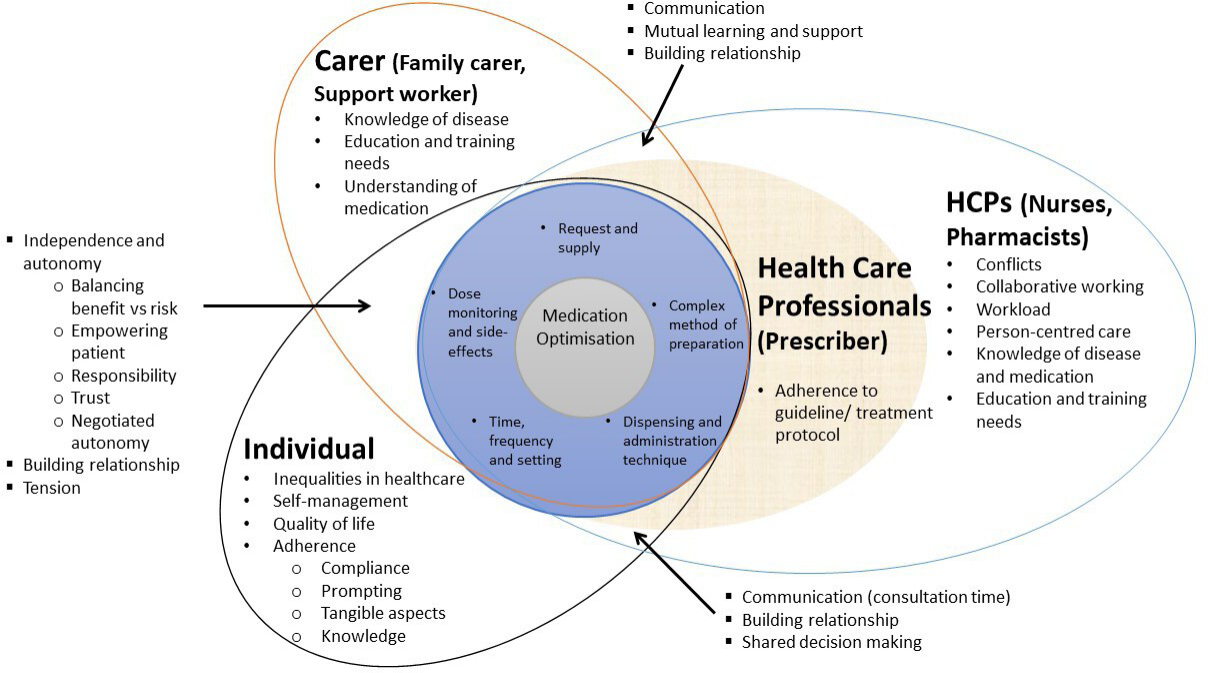
Illustration of subthemes and their interactions with individuals with intellectual disability and their support networks. HCPs, healthcare professionals.

We initially generated a list of 61 codes across 45 included studies. Subsequently, we re-organised them into 35 unique conceptual categories and developed an initial conceptual framework as shown in figure 1. This framework illustrates the crucial roles that carers (including family and other unpaid carers, and paid carers and support workers) and healthcare professionals play in supporting the medication use process for people with intellectual disability and the importance of communication and relationship-building between all people involved. Additionally, we shared and discussed initial findings with the Project Advisory Group and PPI groups to align their lived experiences with our findings, strengthening rigour and shaping the conceptual model.

The identified conceptual categories were then organised under four thematic groups. These were: (1) medication-related issues, (2) navigating autonomy and relationships, (3) knowledge and training needs and (4) inequalities in the healthcare system. The indicator for confidence for each theme, reported in online supplemental material table 2, was measured by the four domains of GRADE-CERQual as described in the Methods section.

### Theme 1: medication-related issues

The first major theme involved practical difficulties related to *acquisition, preparation, use and administration of medications and adherence to a prescribed regimen*. It also encompassed the tension, anxiety and frustration derived from them and potential solutions to mitigate these difficulties.^1719 37 41 43 45^,^54 56 58^

#### Acquisition

People with intellectual disability and their carers may spend a significant amount of time obtaining a complete prescription or the prescribed items. This could be due to complex instructions for ordering medications or a medication shortage. The latter may be aggravated by the need to use a specific brand of proprietary medicine; medications of a specific dosage form (eg, liquid); or unlicensed drugs in some circumstances.^5265^,^69 72 73 77^

Visit to chemist today to collect food supplements, Nutrison and Fortisip. I ordered these items a few days before. Once again the order is not ready … Problem – ordered from wrong supplier. I will have to call again, next time I’m going! Also collected the balance of another med which chemist was short of on my last visit. This happens quite a lot. (family carer; interview)^67^

#### Challenges of preparation, use and administration

Apart from acquisition, the timely *preparation and administration* of a comprehensive list of medications created additional challenges. Studies described excessive efforts from carers in the preparation process to uphold an established routine governing how to prepare and administer medication for individuals. This could be especially true in community settings or for school-age children. The need to crush tablets, mix medication with food or drink for consumption, and the slow pace of food intake due to the person being a young child or having swallowing difficulties increased the demand on the carers further. The complexity of using medication delivery devices also complicates this process, affecting both carers and people with intellectual disability. The unappealing taste, texture, size and smell of the medication could pose additional challenges to successful medicine administration. On this note, the literature also highlighted the added burden triggered by the need to administer additional medications for acute conditions (such as an antibiotic for infections) on top of already difficult medication administration routines.^41 52 55 59 61 62 65 66 68 74 77^

Participant 9: And also practically. If you have 8 residents, and you need to administer medication to 8 residents, for example at 7 am, where you need to prepare and administer all tablets or medications separately. We also have children that need 10 or 12, sometimes there are also capsules and also syrups and… Participant 7: And drops. (care professionals; focus group)^77^

Three participants mentioned disliking the taste of the medication and one spoke about the fear of choking on the tablet. (people with intellectual disabilities; interview)^41^

I used to have that (Accuhaler), but it was a bit hard to use. (person with intellectual disability; interview)^61^

These issues not only presented practical difficulties but also created emotional burden for people with intellectual disability and their carers.

#### Challenges in adhering to prescribed schedule and strategies to overcome them

Medications may not be taken according to the prescribed schedule due to a lapse in memory, changes in routine or intentional non-adherence because of lack of perceived benefit, side effects and/or interference with social activities. The individual’s reluctance to use medication in a public place and perception of lack of desired effects despite having good compliance with medication contributed towards issues in adherence.^1941 46 48 51^,^53 55 58 61 65 66 68 69 72^

It’s good, but I don’t like doing it when I’m around… when I’m with people. (person with intellectual disability; interview)^61^

Half of the participants spoke of wanting to change or stop their medication, and two participants reported wanting to take a different tablet, as the current medication was not having an effect in controlling their negative behaviour and was not living up to the participant’s expectations. (people with intellectual disabilities; interview)^41^

The authors of studies, however, reported a number of strategies to assist medication adherence using: (1) reminder alarms and visual cues, (2) multicompartment compliance aids, (3) direct observation of medication self-administration and (4) patient education.

I put this one [Tablet] on the kitchen table; the orange one is beside my bed. (people with intellectual disability; interview).^61^

#### Monitoring and communication of intended and unintended effects

Another important concept identified was monitoring and communication of both intended and unintended medication effects. Individuals and their carers reported concern of overmedication and associated effects. They also identified how polypharmacy could produce side effects through drug-drug interaction, underscoring the necessity to engage in effective communication with HCPs to review the medication regimen once side effects were observed.^1719 37 40 41 43 45 47 49^,^51 53 56 58 60 61 63 64 66^

It is a shame how much they affect my son. When starting new medications he is very drowsy and we ‘lose’ the real him. He becomes a shell. We had to rapidly increase his dose of phenobarbitone to try and get his seizures under control and he was so heavily sedated as a result it was heartbreaking to see him. He was lifeless almost. It is sad to think that in order to control the seizures, you have to use a medication that takes a piece of them away almost. (family carer; interview)^79^

Using the medication is tough…They said you have to be prepared for side effects, but your body will get used to it. Anyway, it takes quite a long time [to get used to], and I have a lot of problems with the medication. (person with intellectual disability; interview and focus group)^40^

We had someone that was prescribed medication and, actually it was interacting and made that person really unwell …… that there was this side effect and phoned the GP …… and immediately the medication was stopped. (service manager; focus group)^17^

However, a potential challenge for people with intellectual disability was to recognise and communicate side effects when they occurred. Whether this was feasible depended on their age, their communication skills, the severity of intellectual disability and whether they had been provided with suitable information and education or had someone who was able to recognise unusual symptoms or behaviours following the administration of the medication and communicate with HCPs on their behalf.

It’s very hard, the side effects, [child] can’t tell us. We’re guessing for him. I mean some can give you headaches. You’ve to look out, you know we wouldn’t know whether he has a headache. He can’t tell us. Nausea, he’s never ever vomited. So if he was to go onto a new medicine and he vomited, I would say “right, that’s the new meds”. (family carer; interview and free text analysis)^66^

…Rather than memorising that zuclopenthixol can cause this, this and this, and lithium can cause this this and this… I tend to look at how the person is… and then, if he’s got a problem, you look back at the medication and see if it could be related to it and explore it then. (carer or support worker; interview)^47^

### Theme 2: navigating autonomy and relationships

The second major theme was the context in which medication support was provided. This core theme involved the aspects of independence, autonomy and conflicts between the carer and the person with intellectual disability. These factors had a significant influence on medication usage. Positive relationships between a person with intellectual disability and their carers promoted shared decision-making and person-centred care, and prevented problems related to medication use.^1719 37 39^,^50 53^

#### Independence, autonomy and conflicts

Some people with intellectual disabilities might lack the capacity, as perceived by their carer and HCPs, to handle medication and to make decisions about medication. In such circumstances, carers or HCPs took responsibility and acted on their behalf.^3739^,^41 43^

Because he looks OK there is an assumption by them that he fully understands sometimes complex sentence structures. It would help if medics were aware of this level of capability and to adjust their conversation appropriately. (family carer; interview)^79^

I don’t think [a particular service user] could really give a reliable response to questions like “do you think your current medication is right for you?” So I don’t think he can be involved in making those [medication] decisions. (paid carer; interview)^39^

She would never be able to learn it herself—to inject insulin—she is too dependent on us for that. To ﬁne-tune the doses, there are too many operations needed, she could never do that. (social worker; interview)^54^

Nevertheless, when a person with an intellectual disability had capacity to make decisions or was able to be involved in decision-making about medication, including the administration of medication, their preference and right to autonomy might be overlooked or disregarded.

However they also revealed the frustration they experience when doctors talked to the caregiver rather than directly to them: ‘‘I always have to ask Y, cause the doctors explain it to her[carer]”. (person with intellectual disability; interview)^61^

But nobody asked them [people with intellectual disabilities] if they are happy or not. (support staff; focus group)^17^

Oftentimes, care staff dictate and control the individuals, not allowing them to make decisions for themselves and expecting them to be perfect and well behaved 100% of the time. (HCP; interview)^37^

In certain instances, caregivers found themselves taking on the role of ‘lifestyle police’^75^ when, based on their judgement, people with intellectual disability were unable to act accordingly, to what others considered to be in their best interests. This could create conflicts between carers and people with intellectual disability when trying to manage disease and make decisions about medication use.

However, mutual trust building, embracing the notion of balancing risk and benefit were helpful for empowering the person with intellectual disability to make their own decisions not only for medication use but also for managing the co-occurring condition by healthy lifestyle choices.

… if she chooses not to take that that’s up to her […]We’ve only had, like she said, one incident where she has chosen not to and then she did come back down later on and take it but it’s just about giving people the option and also saying to her ‘these are the consequences of you not taking your medication and it’s entirely up to you. (care manager; interview)^50^

She wanted to go to the village by herself. Previously, this was not allowed because she sometimes stole food from the shops. … We made a plan, practiced together and succeeded. Now she does some shopping for us, and buys her own healthy desserts. Sometimes it goes wrong, then we talk about it. She is so proud of doing this. (social worker; interview)^54^

#### Building relationships, person-centred care and shared decision-making

The conflicts arising from different perceptions of capability to make ‘correct’ decisions and the need for relationship-building extend beyond the person with intellectual disability and their carers to include HCPs and other care and support personnel. Building positive relationships between all members of a person’s support network underpinned effective medication assistance. When considering medication withdrawal, the need for a good relationship was seen as important for its success.^1719 39 40 42 44^,^50 54 56^

All went well in agreement, I could always phone or come for consultation. (person with intellectual disability; interview)^56^

So, I can see that, you know, working together with everyone, the staff, the family, it might not be only the medication being given but maybe alternative medication can also be given to help them, while the medication is being withdrawn. (service manager; interview)^17^

Authors of included studies vividly emphasised s*hared decision-making* in determining the treatment and medication regimen for a person with intellectual disability. This collaborative approach could involve the individual, paid and unpaid carers, HCPs and other members of a multidisciplinary team who supported the individual. It was the case that different people contributed valuable knowledge and skills that were required to make optimal decisions.

Although the ﬁnal decision about starting, changing, or discontinuing a medicine is a responsibility of a physician, the nursing staff has an important proactive role in all this, since they often are the ﬁrst to detect and evaluate the possible needs for medical checks of the residents and they arrange any required consultations with physicians. (nurse, interview)^19^

Attending an epilepsy clinic treatment is better IF the neurologist takes time to try [and] understand and talk to the person with ID[intellectual disability] not the carer. (paid carer; interview)^79^

However, in practice, decisions were sometimes made by HCPs without involving people with intellectual disability and their families.

A paid carer ‘raised concern as people with ID [intellectual disability] and their families are often not involved in decision-making about the person with ID [intellectual disability]. They are often informed about the decision after that has been made’.^42^

In addition, tension and conflicts could arise in the shared decision-making process when there are disagreements between different people involved. Such situations were prominently highlighted in decision-making with regard to prescribing of psychotropic medication for challenging behaviour, where psychiatrists often needed to make a difficult choice between either adhering to their professional judgement or settling for a compromise based on family carers’ preference. In the former case, family and carers could be left feeling that their wishes were ignored, while in the latter case the psychiatrists could feel highly pressurised and uneasy.

In the beginning [the psychiatrist] wouldn’t give [any medication]…I was asking them to give her something to get her better because she was stuck and I was suffering too…But [the psychiatrist] didn’t agree with me and gave me a hard time…they didn’t listen to me for three months (FC 08)'. (family carer; interview)^39^

[The family] wanted medication and it’s been a really hard process…we felt that we had to [prescribe] because it was so much what the parents wanted, and we thought if we did that then they would be more on board with looking at the rest of the interventions we wanted to put in place (TP 01). (trainee psychiatrist; interview)^39^

Authors of included studies suggested that ‘tailored’, ‘customised’, ‘individualised’ and ‘person-specific’ care approaches are most effective for optimising medication use among people with diverse levels of intellectual disability, and when the individual had additional health conditions. The tailoring (such as clear and not overly complicated information) would respond to the specific needs of a person with intellectual disability or those who support them and might require constant adjustment depending on the situation.

There could be general diabetes information and then you step it up to more specialised information for the people with ID to meet their needs. (psychologist; interview)^40^

We haven’t had a clear asthma management plan for this client; I guess just having clear instructions for the direct care staff and casuals who might be coming into the house…needs to be very simple… we just need to know what to look out for, what to take and when to call an ambulance. (direct support professional; interview)^62^

This client has epilepsy as well. So if there’s any seizure activity he can get confused and forget things. We’re deﬁnitely more vigilant in checking things when he’s had a seizure. (P21) (direct support professional; interview)^62^

However, providing person-centred care requires additional resources, and in particular, time. This often becomes a burden for carers and HCPs and acts as a barrier.

You just get rushed through these appointments. You have ten minutes, no doctors and nurses have got time to sit and explain diabetes in a way that people with ID [intellectual disability] can understand…they’re always so busy. (residential worker; interview)^40^

### Theme 3: knowledge and training needs

The third theme highlights the barriers to gaining access to information about medication for people with intellectual disability and their carers, along with a lack of specific education and training in optimising medication collaboratively with people with intellectual disability and their carers.^1719 37^,^45 47^

People with intellectual disability expressed a desire for specific information related to their condition and actively looked for relevant information. A shortage of accessible information, tailored information delivery and materials related to disease and/or medication was identified as a problem within the included studies.

Some respondents also reported ‘a lack of accessible information’ for people with ID [intellectual disability] and their carers about diabetes, management and potential complications. Some diabetes and ID [intellectual disability] practitioners described being unaware if accessible information about diabetes existed and where to obtain it. (intellectual disability service practitioner; interview).^40^

However, an unavailability of tailored information materials and training hindered the person becoming independent and making decisions for themselves.

There were two respondents who spoke quite passionately about wanting to be informed about their medication and alluded to it being a ‘right’ to know what was being given to them. (people with intellectual disabilities; interview)^41^

Family and paid carers similarly expressed the importance of accessible information and adequate training related to medications and the conditions being treated that would help them to take the best care of the person with intellectual disability.

The epilepsy nurses gave me lots of information, but not necessarily easy read nothing like that. (family carer, interview)^49^

Medication prescribed but no follow up. No information on possible side effects except the leaflet in the box, and my research on the Internet. No training to instruct me on what to do during a seizure or on how long to wait before calling an ambulance. Depended on word of mouth from other parents and their experiences. (family carer; interview)^79^

The above quotes highlight that carers found it challenging to obtain the required information and resources. As a result, they were sometimes dependent on communication with HCPs or mutual support, which mitigated the unmet information and training needs to some extent. A study showed how shared knowledge and guidance mitigated the issues related to a newly prescribed drug.

I need an efficient pharmacist backing me up. And they’re learning from us, because this latest drug she’s been put on – they’ve never used it before … they’ve given me guidance, advice, help, because I’ve gone to them for it. (family carer; interview and free text analysis)^67^

The included studies mentioned a few educational tools that have been in place in different countries, such as the psychoeducational programme SPECTROM (Short-term Psycho-Education for Carers to Reduce Over Medication of people with intellectual disabilities) which comprised (1) a web-based or paper-based module and (2) face-to-face training.^80^ Participants (paid carers) identified the materials and resources that they appreciated.

… that’s what I took away from your session, the documentation with the medication. Because I loved all of these [Medication resources]. (support worker; interview)^60^

In addition, across studies, there was evidence that familiarity with the disease and medication usage could help reduce the issue of polypharmacy, reduce medication burden and facilitate monitoring of medication-related side effects.

It is a good initiative and made me think now for each patient the long term impact. ……more aware of the risks and more confidence to say no to prescribing…. (prescriber; free text analysis)^43^

…because me myself I am not an advocate of drugs to be honest. If I have a headache I tend not to take Paracetamol, I try to do other things to calm me down. …Camomile tea, because I am aware of some of the side effects of certain drugs on your system and some of them its long term, it builds up and builds up, and then eventually it takes its toll on your system. So it’s just like the drugs that we give to the service users, everyday you go and pop it from the pack and just give it… .its not like a sweet you just give, it’s really dangerous over a period of time. (qualified care worker; focus group)^38^

### Theme 4: inequalities in healthcare systems

In addition to the limited availability and accessibility of tailored information material and training infrastructure, our fourth theme identified further inequalities that people with intellectual disability encountered in the healthcare system. These arose when accessing specialist clinics and there was a shortage of staff who understood their disabilities and had the expertise to address their needs. This created unnecessary obstacles for individuals and their carers to make the best use of medication.^1937 39 40 44 47 55 57 61 64 65 67 69 70 72^,^74 79^

It appears at times that the medical practitioners ‘give up’ on treating people with learning disability and epilepsy, and the individual has to accept that ‘this is their lot’. I wonder whether this would be the same for someone without a learning disability. (care professional; questionnaire survey)^79^

The role of the psychiatrist in the multidisciplinary team was said to be mostly for diagnosing a psychiatric disorder or to give advice on psychotropic drugs. However, the availability of psychiatrists is often a problem. Moreover, organisations face shortages of professional staff, which seems to be an even bigger problem in the outpatient setting. (intellectual disability physician; interview).^57^

In addition to shortages of staff with suitable expertise, people with intellectual disability also often have difficulty in obtaining the required services in a timely manner. Carers needed to ‘fight’ with the system to access the service, although it could take several months, and that was enough to worsen the health of the person.^19^ The challenges also arose, as reported by the authors, when there was a change of doctors or a change in providers such as a transition to group homes from the hospitals.

That is the specialist whom we cannot ﬁnd because she has depression and has been on medication for many years. She was under the care of a doctor who left the [practice] group but he does not take Title 19 anymore. The ofﬁce he left does, but they won’t take his patients. So she was left without a doctor. (family carer; interview)^73^

Furthermore, in some countries, the insurance coverage or cost for providing specialist services for people with intellectual disability restricts available choices for them.

My experience is spending hours on the phone after you spent hours trying to ﬁnd out who is the specialist you can call to see if you can get an appointment. Then what I hear all the time is ‘Oh, you have Medicaid/Medicare, sorry we are out of our quota,’ or, ‘We are not accepting Medicaid patients. (family carer; interview)^73^

#### Overarching concept: ‘Collective collaboration’

In the final stage of the meta-ethnography analysis, further conceptual frameworks were developed to present the ‘line of argument’ through collaboration with the project advisory group and input from the PPI groups.

**Figure 2:**
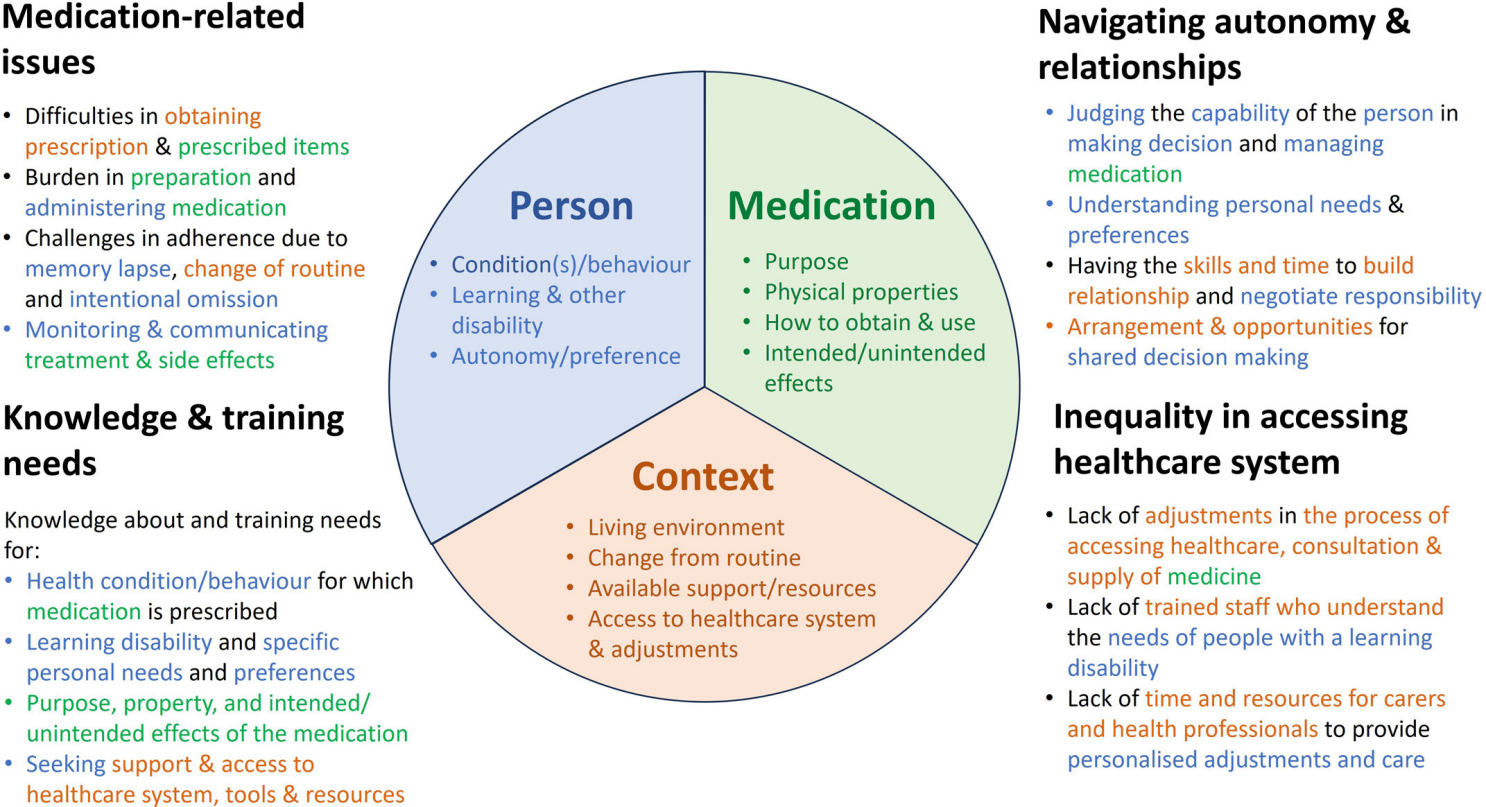
A conceptual framework illustrating how individual conceptual categories within each of the four key themes revolve around the three aspects of ‘person, medication and context’.

The four key themes and associated conceptual categories described above revolved around the multifaceted issues of medication management (figure 1). To effectively overcome the wide range of issues, one needs to adequately understand and address three specific aspects of medication use: the person with intellectual disability, the prescribed medication and the context in which medication is used and managed. Figure 2 illustrates how individual conceptual categories under each theme link to the three key aspects.

While these three key aspects (person, medication and context) of medication use are applicable for any person, there are features unique to people with intellectual disability that require particular attention to ensure that their needs are met.

Understanding a person’s underlying condition(s) and/or behaviour(s) for which medication is being considered or prescribed, along with other health needs and preferences, is the starting point for medication use. These could include a person’s past history related to illnesses, treatments and other life events and circumstances such as poverty and trauma that may have influenced the manifestation of disease conditions, behaviours and medication-related decisions. This information is usually communicated to HCPs by the patients themselves. People with intellectual disability may have communication difficulties which vary according to their degree of intellectual disability; some may have only a few difficulties with spoken language, while others may make use of augmented or alternative communication (eg, symbols) or will have very limited to no communication ability. Good communication with a person with intellectual disability can only occur when prescribers, pharmacists, carers and others have a full understanding of the concerned person’s communication needs. This can be gained by asking those who know an individual well about their specific communication needs. However, collective knowledge about an individual can only be built over time through collaborative working, leading to the formation of positive relationships and improved communication which can greatly reduce problems and enhance the experience of medication use for people with intellectual disability. When people with intellectual disability require others to communicate and advocate for them, carers, nurses and pharmacists play a crucial role as a bridge between them and prescribers.

Simultaneously, it is crucial for people with intellectual disability, their carers and HCPs, particularly those responsible for prescribing, dispensing, storage and administration of the medicine, to possess relevant knowledge about prescribed medications. This includes understanding the medication’s purpose, physical characteristics, as well as its intended and unintended effects. Pharmacists are ideally situated and could play a pivotal role in information sharing and conveying the required knowledge related to medication in this support network.

Lastly, the challenges associated with medication use are closely tied to environmental factors, such as living arrangements, changes in routine, resource availability and healthcare accessibility. Personalised arrangements and adjustments are usually needed to help people with intellectual disability manage their medication use and navigate the healthcare system. Depending on the capability and needs of the individual person with intellectual disability and the readiness or adjustments of the healthcare system to fulfil these needs, someone in the support network may need to advocate and take actions on behalf of the person with intellectual disability. Suitable support in this regard can significantly influence medication adherence and treatment outcomes for people with intellectual disability. Such support needs to be offered at the right time, and thus the better the supporting network, or ‘collective collaboration’ surrounding the person with intellectual disability possesses the collective knowledge, capability and resources that are well coordinated and shared, the more likely that such support will be available when needed to address issues related to medication use. Therefore, in addition to paying attention to personal and medication-related factors, taking a holistic approach within a systems perspective to support an individual would help medication optimisation.

**Figure 3:**
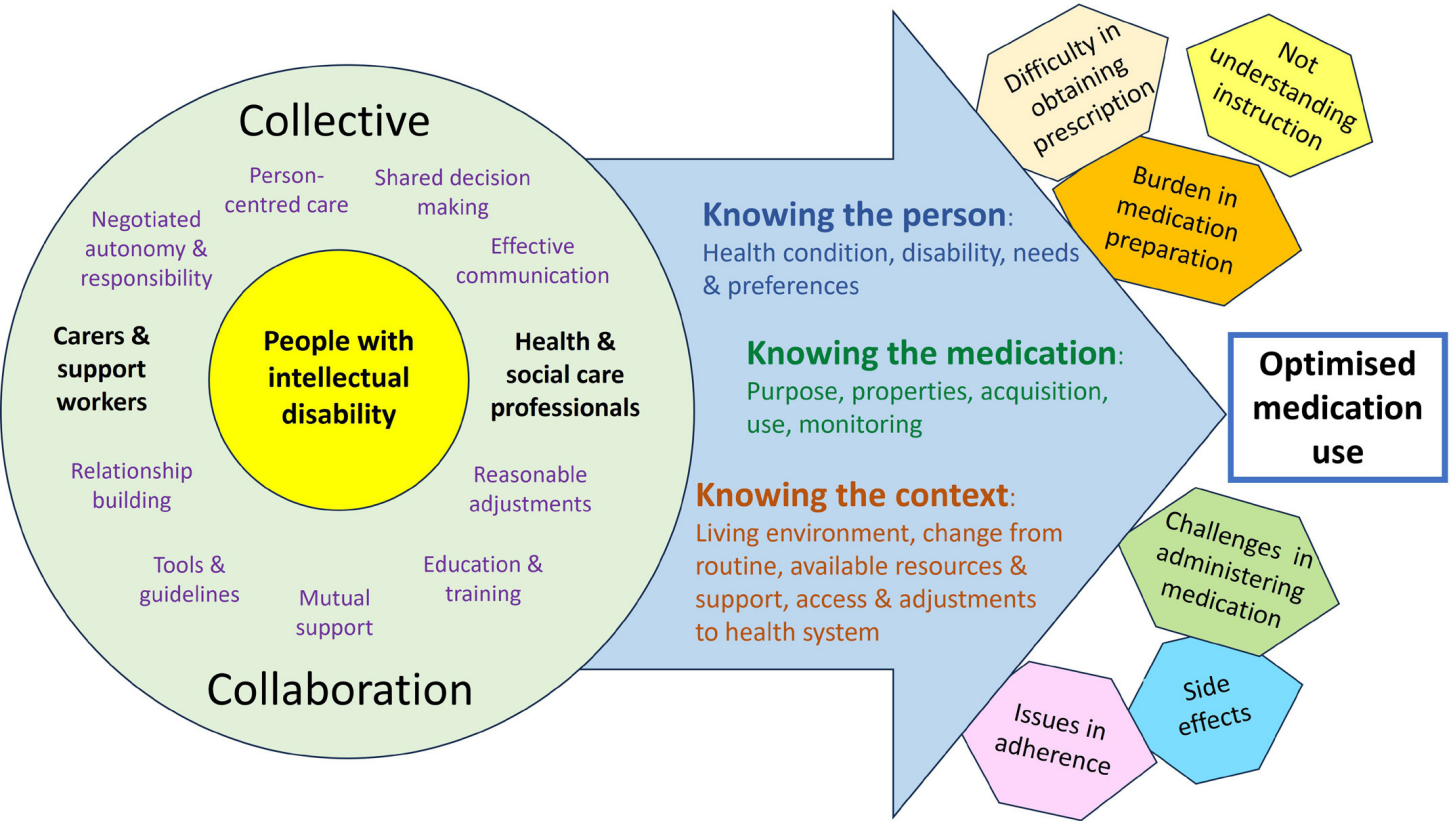
A conceptual model illustrating the overarching concept of ‘collective collaboration’.

Figure 3 provides a diagrammatic presentation of our line of argument and the overarching concept ‘collective collaboration’, derived from the themes emerged from the literature and summarised in the prior conceptual models as well as our understanding of medication use process gained from the literature and the experience shared by our PPI groups. The model is underpinned by the four major themes and the key aspects of ‘person, medication and context’. The integration of evidence across studies, as presented above, indicated that having an excellent understanding of the person with intellectual disability, including their disabilities, preferences and communication needs, their contexts and resources; the medication and the conditions or behaviours for which the medication is prescribed; and the ability to make necessary adjustments to meet personal needs are all required for medicines optimisation. A joint effort between people with intellectual disability and those forming the collective supporting network including prescribers, family and paid carers, pharmacists and other HCPs, through person-centred care, shared decision-making, effective communication and mutual support is necessary to achieve this aim. It may be worth highlighting that while these proposed solutions are supported by the qualitative evidence that we synthesised and also echoed by the experience of our PPI groups, formal testing of their effectiveness will be needed to validate our line of argument.

## Discussion

This is to our knowledge the first qualitative evidence synthesis examining the experiences and challenges at various stages in the medication use process and across a broad range of medications by people with intellectual disability and people who support and/or care for them. A rigorous approach using meta-ethnography was adopted to derive evidence from 45 primary studies, leading to the development of an overarching concept of ‘collective collaboration’ to promote effective medicines optimisation with people with intellectual disabilities. A key strength of our study is the continuous involvement of individuals with lived experience, which added depth, rigour and trustworthiness to the synthesis. The findings of this meta-ethnography corroborate with the findings from other systematic reviews with narrower scopes,^22^,^2581^ which highlighted the challenges for people with intellectual disability to understand the medication that they are taking, the training needs for people supporting them and specific challenges faced by those with diabetes and their carers managing the disease and their medications. Our meta-ethnography extends this literature by synthesising additional evidence related to prescribing decisions particularly in relation to use of psychotropic medications for challenging behaviours and other issues spanning across acquisition, preparation, administration and adhering to prescribed medication and monitoring and communicating treatment effects including side effects. We drew together the diverse issues in the medication use process, highlighted the interconnectedness and intricate relationships between people with intellectual disability and different people playing a part in their supporting network to create the line of argument of collective collaboration which centred around people with intellectual disability, and which needs to cover the knowledge, capability and resources required to ensure optimal medication use.

While the advantages of collaborative work and the need for shared decision-making, person-centred care and reasonable adjustment featured strongly in the qualitative evidence that we synthesised and have also been widely acknowledged in the literature related to medication use for people with intellectual disability, collaboration tailored to the needs and specific care settings at the individual level is often lacking in practice. Consequently, it may be worth pointing out that the model of care and possible ways to overcome barriers for optimised use of medications that we depict in figure 3 may represent an ideal and yet theoretical scenario at present. There remains a theory-practice gap which needs to be addressed through theory-driven design and empirical testing of interventions and service organisation. The conceptual models that we created in this meta-ethnography provide a comprehensive and yet reasonably simple overview of possible issues and potential solutions, which we hope will facilitate the design and testing of future supporting strategies, care systems and policies.^22 82 83^

A few issues are worth highlighting. First, shared decision-making with people with intellectual disability and their carers is vital while recognising that information about any decision must be provided in a manner that is most appropriate to help someone with intellectual disability understand. However, some people with intellectual disabilities will lack capacity to make decisions about medication and the Mental Capacity Act, 2005 in England and Wales must be followed. Other countries have similar legislation, for example, the Adults with Incapacity (Scotland) Act, 2000. Further work is needed to thoroughly understand how legislation governing capacity and decision-making may affect medicines optimisation within different jurisdictions. In addition, personal factors such as an individual’s circumstances and life history—particularly experiences of poverty or trauma—can also influence medication decisions and illness presentation, complicating personalised care. In terms of integrative care incorporating ‘collective collaboration’ that is described in our conceptual models, multidisciplinary involvement (in addition to prescribers, pharmacists and nurses) such as inputs from behavioural therapists, psychologists and members of staff from education institutions and social services is increasingly common and could be very important to ensure a holistic approach. These may not have been sufficiently featured in our conceptual models and should be further integrated into future revisions and refinement of the conceptual models as required.

The perspectives of children and younger people with intellectual disability were notably under-represented in the studies included in our meta-ethnography. In addition, the included studies were exclusively conducted in Western, high-income countries. The ethnicity of the participants was infrequently described, and where reported, tended to be predominantly white. The generalisability of our findings to children and young adults, and to communities and countries with predominantly non-white ethnic origin may need to be further explored, as medication use could be influenced by ethnic and cultural factors. Furthermore, the predominant focus of previous studies on psychiatric medications overlooked other important medication use issues, such as pain management, obesity and constipation, further limiting applicability across patients with different healthcare needs.

While we applied the GRADE-CERQual (online supplemental material 2) framework to assess the robustness of our findings, it is essential to acknowledge the ongoing discourse surrounding the evaluation of qualitative research.^35^ First, focusing solely on methodology might overlook valuable insights leading to debates over the definition of a good qualitative study. Although we conducted a CASP assessment to gauge and identify the *methodological limitations* of individual studies*,* this approach does not help us to appraise the significance of the themes used in the line of argument. Second, we have used the number of studies supporting each theme as a proxy for coherence. However, this approach does not justify the development of a line of argument as we have included all relevant evidence, including those appearing to be exceptions, into our line of argument. Third, we somewhat reluctantly counted the number of codes supporting a theme as a proxy for adequacy, but it does not quite fit with how we interpret the line of argument. Giving equal weight to each code implies that more studies always make a finding more valid, which is not necessarily the case. Finally, GRADE-CERQual emphasises distinguishing different levels of data relevance (direct, partial, indirect or not), but the included qualitative studies are more focused on the specific context of individual primary studies and data captured from a small number of participants. These studies might not appear to be directly relevant to the review’s research topic. As a result, our themes were extracted from the core ideas from a variety of distinct contexts.

This meta-ethnography has a number of limitations. As the research question that we were trying to address was very broad, we might not have covered and described all specific issues in the medication use process for people with intellectual disability, for example, issues related to communication of information between different groups of people involved or issues associated with transition between different services and care settings. In addition, clinical practice and service organisation, including multidisciplinary teamwork, are continuously evolving and improving. The studies that we included, and hence our findings derived from the evidence that they reported, might not have sufficiently captured recent developments in integrated care for people with intellectual disability, such as the increasing involvement of behaviour therapists and psychologists mentioned above. Nevertheless, we hope the overarching concept and key themes and aspects of care described in our line of argument and related conceptual models are sufficiently comprehensive to be applicable across diverse scenarios. Further work to explore and explicate how different contexts (eg, community vs residential settings; different age groups and people with different severity of intellectual disabilities; and during transition between services) impact on the manifestation of different issues and the needs for different solutions would be invaluable. Further development and refinement of our conceptual models to reflect evolving configuration and delivery of health and social care will also be needed. We examined and interpreted the literature mainly through the lens of UK healthcare settings, potentially overlooking issues relevant to non-western nations (in particular, below the Brandt line) or high-income countries with very different healthcare systems and service delivery (eg, USA, where health insurance is pivotal).^84^ This could limit the generalisability of the conceptual model. Additionally, the absence of explicit reporting on the severity of intellectual disability among included populations may have further limited the coverage of medication use for individuals with severe or profound intellectual disabilities. While it is possible that capturing the perspectives of individuals with more complex communication or cognitive challenges remains a methodological difficulty in the literature, their under-representation poses a gap in current knowledge. Lastly, our findings are constrained by the available literature, potentially omitting insights into issues related to cultural and ethnic disparities, as well as variations in healthcare settings such as hospitals and educational institutions which were not well covered in the studies meeting our inclusion criteria. Despite these limitations, the research highlights the critical role of ‘collective collaboration’ among patients, carers and health and social care providers in improving medication adherence and optimising pharmacotherapy for people with intellectual disability. We encourage translating this conceptual model of ‘collective collaboration’ into practical application by following the principles and approaches highlighted in the model, particularly in settings in which medication use is complex and ongoing, such as residential care facilities, where a coordinated effort among care providers with involvement of people with intellectual disability at the centre of care may promote medication optimisation. Furthermore, this approach underscores the importance of a tailored, holistic support system to address the unique needs of this group.

In conclusion, effective medication management for people with intellectual disability requires a collaborative, person-centred and holistic approach. Prioritising shared decision-making, offering both educational and practical support, and building strong, trusting relationships among all stakeholders can foster a strong and supportive care network. The principles of this collective effort could guide the design and evaluation of strategies to enhance optimised use of medication and promote better therapeutic outcomes among people with intellectual disability.

## Supplementary material

10.1136/bmjopen-2024-090876 online supplemental file 1

## Data Availability

All data relevant to the study are included in the article or uploaded as supplementary information.
