# Supplementary material for: Challenges of using and managing medication: a meta-ethnography of the experiences and perceptions of people with intellectual disability and people who support them
Source: BMJ Open. 2025 Sep 18;15(9):e090876. doi: 10.1136/bmjopen-2024-090876 (PMC12458619; doi:10.1136/bmjopen-2024-090876)
Supplement: online supplemental file 1 [file bmjopen-15-9-s001.pdf]

The challenges of using and managing medication: A meta-ethnography of the experiences and perceptions of people with intellectual disability and people who support them.

Supplementary material 1: MEDLINE search strategy

Ovid MEDLINE(R) ALL <1946 to September 19, 2022>
Date searched: 20/09/22
1 Developmental Disabilities/ or exp Learning Disabilities/ or Persons with Mental Disabilities/ or exp Intellectual Disability/ 145255
2 Neurodevelopmental Disorders/ or exp Child Development Disorders, Pervasive/ 48688
3 ((learning or intellectual* or developmental* or neurodevelopmental*) adj (disabilit* or disabled or handicap* or impair* or retard* or deficien* or disorder* or subnormal*)).kf,tw. 69499
4 (mental* adj (disabilit* or disabled or handicap* or impair* or retard* or deficien* or subnormal*)).kf,tw. 44788
5 (development* adj1 delay*).kf,tw. 21252
6 (down* syndrome or fragile x or william* syndrome or angelman or cri du chat or smith magenis or de lange syndrome or rubinstein taybi or prader willi or patau* syndrome or trisomy 13 syndrome or wagr syndrome* or wilms tumo?r aniridia).kf,tw. 41264
7 "profound intellectual and multiple disab*".kf,tw. 124
8 (PMLD or PIMD).kf,tw. 265
9 (autis* or asperger* or neurofibromatos* or hypothyroid* or phenylketonuria or digeorge or lesch nyhan or rett* syndrome or overgrowth syndrome* or pervasive development* disorder* or f?etal alcohol or prenatal alcohol exposure or fasd or velocardiofacial or velo cardcregio facial or velo cardiofacial or velocardio facial or klinefelter* or childhood disintegrative or static encephalopath*).kf,tw. 141652
10 (22q11?2 adj1 deletion).kf,tw. 1842
11 learning difficult*.kf,tw. 2737
12 1 or 2 or 3 or 4 or 5 or 6 or 7 or 8 or 9 or 10 or 11 [LDs including neurodevelopmental, "learning difficulties" and specific named conditions that can be associated with LDs] 339101
13 exp Medication Adherence/ or Self-Management/ or Medication Review/ or Medication Therapy Management/ or Deprescriptions/ or Inappropriate Prescribing/ or Polypharmacy/ or Self Administration/ 53707
14 ((medication? or medicine?) adj support).kf,tw. 194
15 ((medication? or medicine? or drug?) adj4 (adheren* or nonadheren* or persisten* or complian* or noncomplian*)).kf,tw. 34815
16 ((medication? or medicine? or drug?) adj3 (understand* or knowledg*)).kf,tw. 14681
17 ((medicine? or medication?) adj management).kf,tw. 5034
18 ((medicine? or medication?) adj review?).kf,tw. 2865
19 (selfmanagement or self management).kf,tw. 24772
20 ((medication? or medicine?) adj3 (administ* or selfadminist*)).kf,tw. 13297
21 ((medication? or medicine? or drug? or prescri*) adj2 (optim* or appropriate* or inappropriate*)).kf,tw. 23238
22 ((medication? or medicine? or drug?) adj1 (discontinu* or taper* or withdraw* or reduction or reducing or decrease or decreasing)).kf,tw. 13077
23 (deprescri* or de prescri*).kf,tw. 1798
24 (overprescri* or over prescri* or underprescri* or under prescri*).kf,tw. 2578
25 polypharmacy.kf,tw. 10186
26 ("medication? use" or "medication? usage" or "medicine? use" or "medicine? usage").kf,tw. 24420
27 ((medication? or medicine?) adj (reminder? or list? or information)).kf,tw. 3410
28 ((medication? or medicine? or drug?) adj3 decision making).kf,tw. 1494
29 Pharmacists/ or "pharmacist*".kf,tw. 44850

30 "Off-Label Use"/ or off label.kf,tw. 11455
31 13 or 14 or 15 or 16 or 17 or 18 or 19 or 20 or 21 or 22 or 23 or 24 or 25 or 26 or 27 or 28 or 29 or 30 232045
32 12 and 31 1949
33 exp animals/ not humans/ 5048617
34 32 not 33 [A: full search: un-filtered] 1904
35 cochrane database of systematic reviews.jn. or search.tw. or meta analysis.pt. or MEDLINE.tw. or systematic review.tw. [High precision systematic review strategy from Wong 2006 / Montori 2005] 636140
36 "systematic review".pt. 207500
37 35 or 36 [Systematic reviews filter] 644304
38 ((("semi-structured" or semistructured or unstructured or informal or "in-depth" or indepth or "face-to-face" or structured or guide) adj3 (interview* or discussion* or questionnaire*)) or (focus group* or qualitative or ethnograph* or fieldwork or "field work" or "key informant")).ti,ab. or interviews as topic/ or focus groups/ or narration/ or qualitative research/ [Qualitative studies filter from Uni of Texas Health, as tested by Wagner 2020] 483759
39 34 and 37 [B: SRs filter] 141
40 34 and 38 [C: Qual filter] 121
41 34 not (39 or 40) [D: neither filter] 1653

Search strategies and filters from the following studies were consulted in development of the MEDLINE search strategy:

- Sheerin F, Eustace-Cook J, Wuytack F, Doyle C. Medication management in intellectual disability settings: a systematic review. Journal of Intellectual Disabilities 2019;25(2):242-76. http://dx.doi.org/10.1177/1744629519886184
- Morel T, Nguyen-Soenen J, Thompson W, Fournier J-P. Development and validation of search filters to identify articles on deprescribing in Medline and Embase. BMC Med Res Methodol 2022;22:79. https://doi.org/10.1186/s12874-022-01515-x
- Adams D, Hastings R, Maidment I, Shah C and Langdon P. Deprescribing psychotropic medicines for behaviours that challenge in people with intellectual disabilities: a systematic review. [unpublished; personal communication].

The following tools were used to identify search terms and refine the MEDLINE search strategy:

- Systematic Review Accelerator SearchRefinery. https://sr-accelerator.com/#/searchrefinery
- Searchrefiner: a query visualisation and understanding tool for systematic reviews. Proceedings of the 27th ACM International Conference on Information and Knowledge Management. 2018 Oct 17:1939–42.
- Sinclair S, Rockwell G. Voyant Tools. 2016. http://voyant-tools.org/

The MEDLINE search strategy was peer reviewed by Andy Hickner, Education and Outreach Librarian, Weill Cornell Medicine, using the PRESS Checklist:

- PRESS Peer Review of Electronic Search Strategies. Last updated September 26, 2022. https://www.cadth.ca/press-peer-review-electronic-search-strategies-0

Supplementary material 2: Description of Project Advisory Group and Patient and Public Involvement (PPI) Groups

Our Project Advisory Group (PAG) was established ad hoc as part of the planned project. The PAG consisted of people with intellectual disabilities, staff members from charitable organisations, and healthcare professionals with expertise in supporting people with intellectual disabilities, including a clinical psychologist, a psychiatrist, a general practitioner, a pharmacist and a nurse. Many of the PAG members were also senior academics with research expertise in this subject area. The chair of the PAG and other group members were all familiar with communicating with people with intellectual disabilities. Appropriate adjustments were made such as avoiding jargons and actively inviting members who were expert by experience to contribute their opinions in the group meetings. Members of the PAG were involved in the project from the outset, contributing to the development of the grant application and providing ongoing oversight of the project. The PAG reviewed and discussed emerging themes and early versions of conceptual models developed by the research review team, and provided comments that directly informed the refinement of the conceptual models.

In addition to the PAG, two patient and public involvement (PPI) groups were also planned and formed as integral parts of the broader evidence synthesis project. One of the PPI groups included people with intellectual disabilities. Meetings of this PPI group were facilitated by the project's PPI lead, who had substantial experience in working with people with intellectual disabilities as a project manager in a charitable organisation. The PPI group meetings were conducted virtually, allowing participants to remain in environments that were familiar to them. PPI group members with intellectual disabilities were supported by workers who have been supporting them outside the research project during (or sometimes before/after) the meetings. Regular breaks were built into the sessions to accommodate participants' needs and to ensure their comfort and engagement. The second PPI group consisted of family carers and health and social care professionals who support people with intellectual disabilities. Meetings for this group were conducted separately to gather their perspectives. Three charitable organisations with established experience and expertise in supporting and/or advocating for people with intellectual disabilities were contracted as collaborators of the research project to facilitate recruitment of PPI group members and to provide ongoing support for their participation in PPI group activities. We deliberated created two groups to ensure that people with intellectual disabilities were able to freely share their views and experience without being inhibited by the presence of their family carers and health and care professionals. The project PPI lead coordinated communications with PPI group members, facilitated group meetings, supported group members' participation and ensured their engagement throughout the project.

As described above, the PAG comprised of individuals representing key stakeholders with extensive experience

working with people with intellectual disabilities and their carers as well as expertise in conducting research in this field. The PAG played a vital role in shaping the research questions, refining the methodology, and offering expert advice and guidance throughout the broader project. By contrast, the two PPI groups made contribution towards the meta-ethnography and the border project mainly by sharing their lived experiences, helping the research review team to become sensitised to issues of importance to them (as service users) and to contextualise and relate the research findings to real-world practice. Through regular meetings and discussions during the synthesis phase, their contributions were instrumental in highlighting important themes (such as shared decision-making and reasonable adjustment while providing person-centered care) and in identifying areas of concern that appeared to be underrepresented in the existing research literature. Additionally, the PPI groups played a key role in developing an easy-read version of the meta-ethnography's key findings, which aimed at making the results more accessible to a wider audience.

As we were working with rather than doing research on PPI group members, the research review team found inputs from the PPI groups very helpful in enhancing research reviewers' understanding of evidence reported in the literature and in contextualise and validating the review findings. We did not encounter disagreements in perspectives between the PPI groups and the researchers. A few suggestions made by the PPI groups fell outside of the scope of our project, but we were able to incorporate them as recommendations for further research for the wider project. Findings of the meta-ethnography were shared with the PPI groups as part of our efforts to acknowledge and honour their valuable contributions to the research, and PPI group members were generally in agreement that the findings and conceptual frameworks derived from them reflected their experience and concerns related to medication use.

Supplementary material figure 1: PRISMA flow diagram illustrating the study selection process

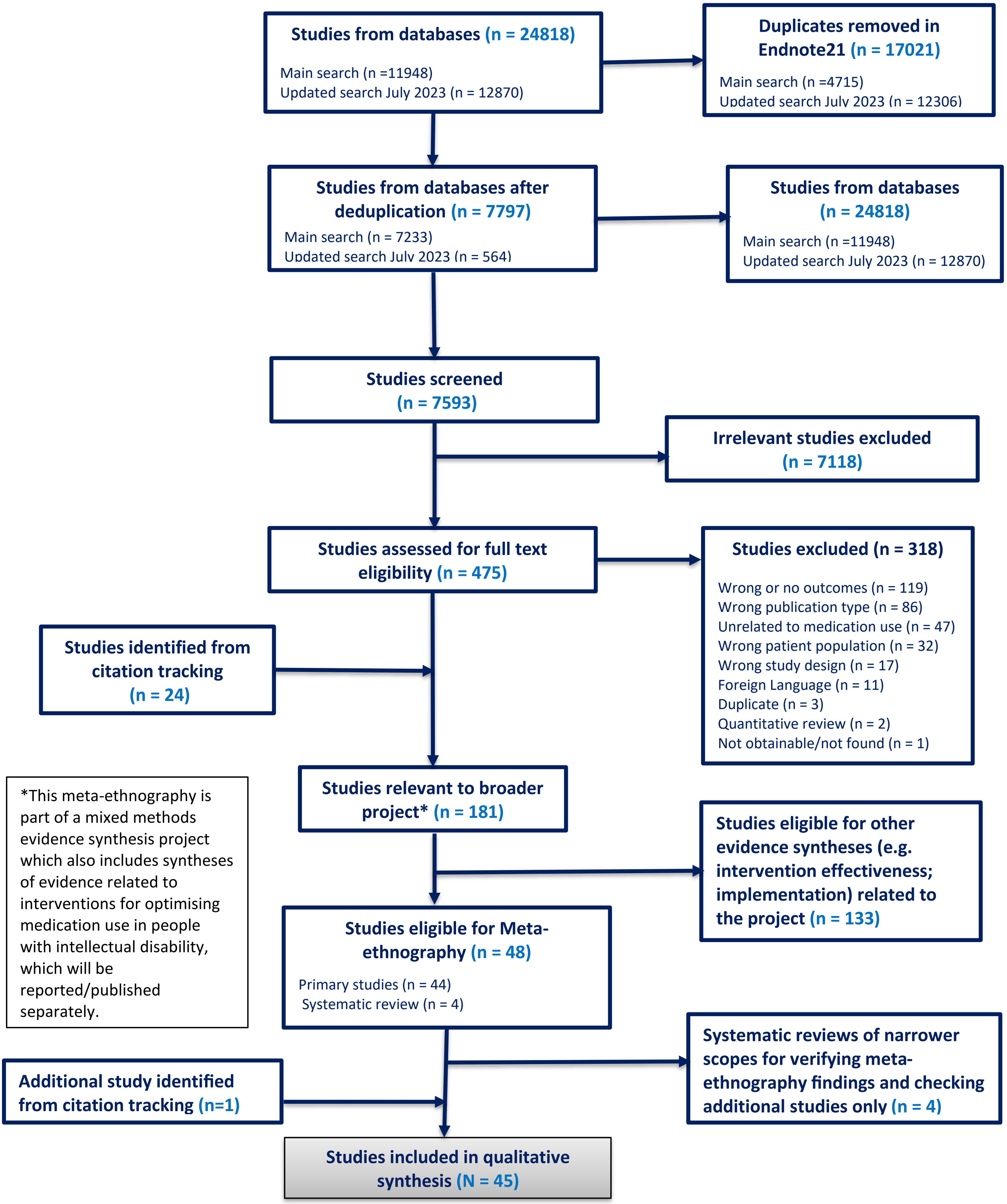

Supplementary material
Table 1: Characteristics of the included studies

Author, year; Country	Setting	Data collection	Population; Number	Description of the condition and/ Medication *(as reported in the primary studies)*	Study focus
Barratt *et al* 2023[1], Australia	Community care home	Semi-structured interviews	Support worker (Disability support worker, house managers). n=10	Adults with complex needs	Perceptions on pilot SPECTROM training programme, including content, delivery and context, and its appropriateness in an Australian context.
Both *et al* 2018[2], The Netherlands	Community setting (Recruited via the Dutch Foundation for patients with Tuberous sclerosis complex [TSC] and health care professionals [HCPs])	Semi-structured interviews	Parents and people with intellectual disability. n=28 (16 people with intellectual disability and 12 family carer)	Young adults with mild to severe intellectual disability and confirmed diagnosis of Tuberous sclerosis complex (TSC- confirmed either by genetic testing or based on the diagnostic criteria)	The study reported the concerns and care needs of young adult patients with TSC in medical, psychological, and socioeconomical domains.
Brown *et al* 2017[3], Scotland	Multiple setting; social care community residential service, primary and secondary diabetes services, Health service area	Semi-structured interviews	Diabetes practitioners, specialists from intellectual disability (ID) teams and support workers. n=29	People with intellectual disabilities who have diabetes	Perceptions and experiences of health and social care practitioners caring for people with intellectual disability who have diabetes and identify their education needs and service development opportunities.
Caoili *et al* 2023[4], USA	Not specified (Community setting-Recruited through flyers, emails, and social media posts, across USA)	Focus groups	Multiple (people with intellectual and developmental disability and mental healthcare needs, family caregivers, prescribers, and non-prescribing mental health professionals). n=43	Adults with intellectual and developmental disabilities (IDD) and needs mental healthcare support.	The themes from the focus group feed into the development of a tool for health care providers to use as a guide to address the complex needs

Cardol *et al*. 2012a[5], The Netherlands	Small community houses and residential facility	Semi-structured interviews	Professional carers. n=13	People with mild to moderate disabilities and diagnosed with diabetes	The role of professional caregivers in providing support to people with a mild or moderate intellectual disability (ID) who have diabetes.
Cardol *et al*. 2012b[6], The Netherlands	Small community houses and residential facility	Interviews	People with intellectual disability n=17	People with mild to moderate disabilities and diagnosed with diabetes	Perception of Diabetes and self-management of people with intellectual disability (ID)
Crossley and Withers 2009[7], UK	'Ordinary' community houses	Semi-structured interviews	People with intellectual disability n=8	Adults with mild to moderate intellectual disabilities and taking long-term anti-psychotic medication	Explored the knowledge and understanding of people with intellectual disability
Davis *et al* 2015[8], Australia	Residential sites providing around-the-clock access to support	Semi-structured interviews	Direct support professionals (DSPs). n=22	Adults and young adults with varied level of functional and intellectual abilities with a chronic condition (asthma)	Explored DSPs' experiences and perceptions of asthma management for people with intellectual disability in supported accommodation.
Davis *et al* 2016[9], Australia	Multiple setting (health care, or the offices of relevant support organisations, or the participants' homes).	Semi-structured interviews	People with intellectual disability. n=17	Adults and young adults with varied level of functional and intellectual abilities with a chronic condition (asthma)	Understanding of people with ID about inhaled asthma medication for asthma management
Deb and Limbu 2022a[10], UK	Community setting	Focus groups	Stakeholders involved in supporting with intellectual disabilities (ID)/ autism spectrum disorder (service managers, support staff, trainers, Community Intellectual Disability Team (CLDT) members, psychiatrists, and family caregivers, including an independent advocate). n=26	Adults were prescribed psychotropic medications for behaviours that challenge (BtC) or went through psychotropic medication reduction or withdrawal	Presented data from the outcome of the co-design event day, specifically the discussion and recommendations for developing the SPECTROM module, "effective liaison with the families and advocates".

Deb *et al* 2023a[11], UK	Not specified (Community setting -Recruited via advertisement sent through the UK Voluntary Organizations Disability Group)	Focus groups	Professional carer (Any paid carers, service/home managers, and trainers) n=16	People with intellectual disabilities and prescribed antipsychotic drugs for challenging behaviour	Perceptions of and views on the use of psychotropic medications to address behaviours that challenge (BtC) in people with intellectual disability.
Deb *et al* 2023b[12], UK	Not specified (Community setting -Recruited via email to the UK Royal College of Psychiatrists' Intellectual Disabilities Regional Representatives and Training Programme Directors)	Questionnaire survey (two pen ended questions)	Psychiatrists n=88	People with intellectual disabilities and prescribed antipsychotic drugs for challenging behaviour	Free text data reported UK psychiatrists view and experience of implementing the STOMP initiative
Deb *et al* 2022b[13], UK	Community setting	Focus groups	Direct care staff (support staff, service managers and positive behaviour support (PBS) trainers). n=16	People with intellectual disabilities and prescribed antipsychotic drugs for challenging behaviour	Staff views and attitudes toward medication use for behaviours that challenge with a particular emphasis on the causes of and alternatives to medication for behaviours that challenge
de Kuijper *et al* 2022[14], The Netherlands	Not specified (Community setting - Recruited though participant's clinicians)	Semi-structured interviews	People with intellectual disability. n=7	Adults with mild intellectual disabilities, who are currently undergoing withdrawal of antipsychotic medications	Knowledge about facilitators and barriers to the successful discontinuation of antipsychotic drug use for behaviours that challenge (BtC) in people with intellectual disability
de Weg *et al* 2021[15], The Netherlands	Community setting (sheltered care facilities)	Semi-structured interviews	Intellectual disability physicians (IDPs) n=9	Psychotropic drugs prescribed for people with intellectual disabilities for challenging behaviour	Explore IDPs' expectations and experiences concerning the new Act and the new multidisciplinary guideline with respect to the prescription of psychotropic drugs

Di Blasi *et al* 2006[16], Australia	Community setting (Recruited through invitation letters sent to the owners of community pharmacies in the Department of Human Services Loddon Mallee Region)	Semi-structured interviews	Community pharmacists n=10	People with intellectual disabilities	Pharmacists' role in the provision of healthcare for people with intellectual disabilities, identifying barriers and the strategies and solutions for improving pharmacists' involvement in the delivery of healthcare to this group.
Doyle. C, 2021[17], Ireland	Home setting (within the community)	Interviews and diary notes	Family members (Mothers) n=15	Children with profound to severe intellectual disabilities, prescribed with a variety of medication	Mother's lived experience of giving medication focusing on the range of activities mothers undertake, and how mothers learn about giving medicines.
Doyle. C, 2022a[18], Ireland	Home setting (within the community)	Interviews and diary notes	Family members (Mothers) n=15	Children with profound to severe intellectual disabilities, prescribed with a variety of medication	Mother's lived experience of giving medication focusing on range of activities mothers giving medicines undertake and any other pertinent issues.
Doyle. C, 2022b[19], Ireland	Home setting (within the community)	Interviews and diary notes	Family members (Mothers) n=15	Children with profound to severe intellectual disabilities, prescribed with a variety of medication	Mother's lived experience of giving medication focusing on perceived issues, and knowledge of medication.
Edwards *et al* 2017[20], Australia	Community setting (supported accommodation in Australia)	Semi-structured interviews	Family members (including parents and siblings) n=7	Adults with intellectual or developmental disabilities (ID/DD) taking antipsychotic medication for a range of diseases such as autism, brain injury in childhood/birth, asphyxia, epilepsy, and dementia	Views of parents and siblings of adults with intellectual or developmental disabilities (ID/DD) around the use of psychotropic medication to manage challenging behaviour (CB) where there is no co-existing mental illness.
Erickson *et al* 2016[21], USA	Supported living facilities	Focus groups	Caregivers who are either family or support staff n=30	Adults with intellectual or developmental disabilities(ID/DD) prescribed with a number of medications.	Major issues the caregivers encounter throughout the process of managing medication and how these might be addressed.
Flood and Henman 2021[22], Ireland	Home	Semi-structured interviews	People with intellectual disability, n=6	Adults with intellectual disabilities, prescribed with a number of medications.	Explore the experiences and knowledge of participants with their medications and the medication

Graham *et al* 2020[23], UK	Not specified (Community setting - Participants recruited by Clinical trust members and the interview was conducted at a private meeting room of a hospital)	Semi-structured interviews	Healthcare professionals and carer n=9	People with intellectual disabilities prescribed with at least psychotropic medication	Evaluated the role of the Pharmacists independent prescriber (PIP) from the perspectives of both staff working within the community service and carers who had engaged with the PIP as part of their caring responsibilities.
Gupta *et al* 2021[24], UK	Regional supported living	Interviews and an open-ended discussion	Care professionals (three project managers, two project co-ordinators, and two support workers). n=7	Service users ranged from young adults with acquired brain injuries to older adults with intellectual disabilities.	This paper presents findings from seven one-to-one interviews with care staff who contributed their experiences representing a diverse range of job roles and categories of service-users supported
Hale *et al* 2011[25], New Zealand	Multiple setting; residential care, supported independent living, and independent living	Semi-structured interviews	People with intellectual disability n=14	Adults with mild to moderate intellectual disabilities and diagnosed with Diabetes (type 1 or 2)	The knowledge and understanding of diabetes held by a select group of adults with diabetes
Halmetoja *et al* 2023[26], Finland	24/7 group housing services (three service provider types: one municipal; one private; and one of the third sector run by a trust)	Semi-structured interviews	Nursing staff n=10	People with intellectual disabilities.	Reported the tasks and responsibilities of the nursing staff in the medication management, explored the nursing staff's perspective on what skills and knowledge they are required and the perceptions of nursing staff about the challenges in the medication management process in 24/7 group housing services for adults with intellectual disabilities.
Heslop *et al* 2005[27], UK	Community	Interviews	Multiple (People with intellectual disability, carer, prescriber) n=52	People with a range of age who are receiving psychotropic medication.	Explored what knowledge people with intellectual disability and their carers had about the person's treatment with psychotropic medication.

Higgins *et al* 2015[28], USA	Multiple settings; various types of supported living	Focus groups	Younger adults with intellectual disabilities and their carer n=22 (4 younger adults and 18 carer)	Individuals aged 18–64 years with physical, intellectual/developmental and/or mental health disabilities,	Designing an integrated health and social service program that will better coordinate care for individuals dually eligible for Medicare and Medicaid,
Joos *et al* 2016[29], Belgium	Residential care facilities(RCFs)	Focus groups	Staff members of Belgian RCFs n=24	Individuals with mild to profound intellectual disabilities, who had to administer medication via enteral feeding tube (EFT)	Identified barriers and facilitators experienced by RCF staff members to following guidelines on medication administration via EFT, by conducting focus group interviews.
Jothaprasert 2017[30], USA	Not specified (Community settings- Participants works within community)	Interviews	Mental health professional. n=4	People with intellectual disabilities accessing mental health services	The primary goal is to highlight and dispel any preconceived notion that mental health professionals have toward those with intellectual disabilities.
Lake *et al* 2015[31], Canada	Multiple setting (home and group home)	Focus groups	Parents n=7	Adolescent or young adults with mild to moderate intellectual disabilities and prescribed with psychotropic medication for autism spectrum disorder (ASD), borderline personality disorder, and Bipolar	Parents' perspectives on psychotropic medication use in their adolescent or young adult with autism spectrum disorder (ASD)
Lalor and Poulson 2013[32], Ireland	Not specified (Community settings- Participants work at various supported living settings within the community)	Semi-structured interviews	Care staff n=8	Adults with intellectual disabilities prescribed psychotropic medication	Explored the experiences and attitudes of care staff for adults with intellectual disabilities prescribed psychotropic medication.
Maine *et al* 2017[33], UK	A daycentre or a home/residential setting	Semi-structured interviews	People with intellectual disability n=10	Adults with mild to moderate intellectual disabilities and diagnosed with diabetes (T2DM) between two and 30 years (mean = 9 years).	Qualitatively explored the experiences of people with intellectual disability n=10 self-manging T2D
Marks *et al* 2021[34], Australia	Home	Interviews	People with intellectual disability and parents n=12	Young adults with intellectual disabilities and diagnosed with diabetes (T1DM)	Identified the barriers and facilitators to optimal T1DM self-management for young adults with intellectual disabilities.

Mengoni *et al* 2016[35], UK	Multiple setting: various types of supported living	Semi-structured interviews	People with intellectual disability n=10 and carer n=15	Adults with intellectual disabilities and epilepsy for which they were seeing professionals and/or taking medication	Explored the impact and management of epilepsy in people with intellectual disability
Musselwhite-Knell 2008[36], UK	Social care home	Interviews and focus groups	Care worker n=24	People with intellectual disabilities and prescribed with psychotropic medication	Explored care workers' views on the use of psychotropic medication for people with intellectual disabilities and their role in the administration of it through the use of integrated methods.
Ramerman *et al* 2018[37], The Netherlands	Multiple settings (general mental health care institutions which also provide care and Intellectual Disability service providers' institutions which provide both mental and somatic health care; and support of management)	Semi-structured interviews	Clinicians (Intellectual Disability physicians, psychiatrists, and behavioural scientists). n=11	People with intellectual disabilities and prescribed with antipsychotic drugs	Explored the barriers and facilitators that clinicians experience in implementing and using guideline recommendations on antipsychotic drugs prescription in mental health care for people with an intellectual disability.
Rouse and Finlay 2016[38], UK	Multiple (home, self-advocacy group/residential service)	Semi-structured interviews	People with intellectual disability and supporter n=14	Adults with intellectual disabilities and having autism or down syndrome and co-occurring condition such as diabetes.	Explored repertoires of responsibility in accounts of managing diabetes for adults with ID.
Sandjojo *et al* 2019[39], The Netherlands	Multiple setting; various types of supported living (Raamwerk, a care organisation for people with ID in Noordwijkerhout, the Netherlands.)	Focus groups	People with intellectual disability, legal representatives and support staff members n=37	Adults with borderline to mild intellectual disabilities	Perspectives of people with intellectual disability, legal representatives, and support staff on promoting independence in this group of people (people with ID).

Sheehan *et al* 2019[40], UK	Multiple settings including residential homes, supported living projects or as peripatetic community support workers.	Semi-structured interviews	People with intellectual disability; family carers; paid carers<br>n=38	Adults with mild to moderate intellectual disabilities prescribed with psychotropic drugs for a range of psychiatric disorders.	Explored the experiences and expectations of people with intellectual disability and paid and family carers regarding psychotropic medication use, and how decisions about this are made with healthcare professionals.
Sheehan, 2020[41], UK	Community setting	Focus groups	Psychiatrists (who worked predominantly or wholly with adults with intellectual disabilities in community settings).<br>n=14	Adults with mild to moderate intellectual disabilities prescribed with psychotropic drugs for a range of psychiatric disorders	Explored the influences on making shared psychotropic medication decisions in adults with intellectual disabilities.
Thompson *et al* 2013[42], UK and Ireland	Community settings from the UK and Ireland	Survey questionnaire	Professionals, paid and family carer<br>n=113	People with intellectual disabilities and epilepsy	Explored the impact of epilepsy on individual with intellectual disability and reported the adequacy and quality of treatment available for this group of population.
Trip *et al* 2016[43], New Zealand	Residential or independent living settings	Semi-structured interviews	Key workers (who had worked alongside a person with intellectual disabilities for at least 1 year)<br>n=17	People with intellectual disabilities diagnosed with diabetes	Explored how key workers supported the self-management of diabetes in people with intellectual disability.
Venables *et al* 2015[44], UK	Community care homes; Two NHS trusts in the West Midlands, UK.	Focus groups	Healthcare professionals (medical practitioners, pharmacists and nurses)<br>n=19	People with intellectual disabilities	Explored and presented the problems experienced when prescribing, dispensing and administering oral medicines to children from the perspectives of medical practitioners, pharmacists and nurses.
Whitehead *et al* 2016[45], New Zealand	Residential or independent living settings	Semi-structured interviews	People with intellectual disability and support worker<br>n=31	Adults with intellectual disabilities and a diagnosis of diabetes	Explored the experience and practice of autonomy in relation to the self-management of diabetes for those with intellectual disabilities living in residential or independent living settings and the role of their support workers.

Supplementary material

Table 2: Summary of review findings and GRADE-CerQual assessment.

**Studies contributing to the review findings**	**Methodological limitation**	**Coherence**	**Adequacy**	**Relevance**	**CERQual assessment of confidence in assessment**	**Explanation to CERQual assessment**
1. Medication related issues: Challenges in medication use for individuals with intellectual disability and their carers include prescription issues, preparation, and adherence, particularly in community or school settings. Reasons for non-adherence include taste aversion, side effect fears, and forgetfulness, addressed through alarms and education. Concerns about overmedication and side effects necessitate comprehension of prescribed regimen and vigilant monitoring. Overall, addressing medication obstacles requires a holistic approach covering prescription oversight, adherence aids, and prompt side effect management.						
34 studies [1,2,5,7-9,11,12,14,16-21,23-35,37-42]	Minor methodological limitation [3 studies with serious methodological limitation (regarding research design, recruitment strategy and data analysis)]	No or very minor concern	No or very minor concern	Minor concern [19 studies with direct relevance, 6 studies with indirect relevance and 8 studies with partial relevance related to the context of medication use]	High confidence	Majority of the studies had minor to moderate methodological limitations and had direct relevance to the summary findings. There were no or only very minor concerns for coherence and adequacy.
2. Navigating autonomy and relationships: Medication support for individuals with intellectual disability creates conflicts over autonomy, with carers often assuming controlling roles. Frustration arises when individuals feel excluded from decision-making. Trust-building and shared decision-making empower individuals and improve outcomes. Tailored care approaches are crucial but can burden carers and healthcare providers due to time constraints and discrimination. Overcoming these challenges is necessary for a person-centred approach to optimize medication use in this population.						
41studies [1-30,32-35,37-39,41-43,45]	Moderate methodological limitation [4 studies with serious methodological limitation (regarding research design, recruitment strategy and data analysis)]	No or very minor concern	No or very minor concern	No or very minor concern	High confidence	Only four of the studies supporting the summary findings have serious methodological limitations. There were no or very minor concern for coherence, adequacy and relevance
3. Knowledge and training needs: Individuals with intellectual disability and their carers lack accessible information and training for medication management. They desire knowledge about the conditions and medications, while carers face challenges in understanding and explaining regimens. Issues include overly complex education programs, caregiver- centred communication, and insufficient training opportunities. Mutual learning and provision of information in preferred formats covering disease and medication knowledge						

to reduce polypharmacy and side effects can help. Improving education and training is vital for optimising medication management in this population.						
32 studies [1,3-14,16,18,20,21,23,25-27,29,30,32,33,35,36,38,39,41,42,44]	Minor methodological limitation [3 studies with serious methodological limitation (regarding research design, recruitment strategy and data analysis)]	No or very minor concern.	No or very minor concern.	Moderate concerns [10 studies with direct relevance, 8 studies with indirect relevance and 12 studies with partial relevance]	Moderate confidence	Majority of the studies had for minor to moderate methodological limitations. There were no or very minor concern for coherence and adequacy, However, there were fewer studies with direct relevance compared to the studies with partial relevance.
4. Inequalities in the healthcare system: Individuals with intellectual disability encounter healthcare inequalities due to difficulties accessing specialised clinics and a lack of trained staff. Financial and transportation issues hinder healthy choices, while carers face frustrations with the healthcare system's general approach. These barriers impose unnecessary burdens on medication management for individuals and their carers.						
18 studies[3,6,9,13,15,18,20-22,25-28,30,32,34,41,42]	Moderate methodological limitations [2 studies with serious methodological limitation (regarding research design, and data analysis)]	Minor concern.	Minor concern.	Moderate concerns [4 studies with direct relevance, 7 studies with indirect relevance and 6 studies with partial relevance with the context]	Low confidence	There are limited number of studies with direct relevance to the summary findings; however only two of the relevant studies have serious methodological limitation. There are no or very minor concern for coherence and adequacy.

Supplementary material

Table 3: Quality Assessment of the included studies

Author, Year	Q. 1	Q. 2	Q. 3	Q. 4	Q. 5	Q. 6	Q. 7	Q. 8	Q. 9	Q. 10
Barratt et al 2023[1]	Yes	Yes	Yes	Yes	Yes	Can't Tell	Yes	Yes	Yes	Yes
Both et al 2018[2]	Yes	Yes	No	Can't Tell	Yes	Can't Tell	Yes	Can't Tell	Yes	Can't Tell
Brown et al 2017[3]	Yes	Yes	Yes	Can't Tell	Yes	Can't Tell	Yes	Yes	Yes	Yes
Caoili et al 2023[4]	Yes	Yes	Yes	Yes	Yes	Can't Tell	Yes	Yes	Can't Tell	Yes
Cardol et al 2012a[5]	Yes	Yes	Can't Tell	Yes	Can't Tell	Can't Tell	Can't Tell	Yes	Yes	Yes
Cardol et al 2012b[6]	Yes	Yes	Can't Tell	Yes	Yes	Can't Tell	Yes	Can't Tell	Yes	Can't Tell
Crossley and Withers 2009[7]	Yes	Yes	Can't Tell	Yes	Yes	Can't Tell	Can't Tell	Yes	Yes	Yes
Davis et al 2015[8]	Yes	Yes	Yes	Yes	Yes	Can't Tell	Yes	Yes	Yes	Yes
Davis et al 2016[9]	Yes	Yes	Can't Tell	Yes	Yes	Can't Tell	Yes	Yes	Yes	Yes
Deb and Limbu 2022a.[10]	Can't Tell	Can't Tell	Can't Tell	Yes	Can't Tell	Can't Tell	Can't Tell	Can't Tell	Yes	Can't Tell
Deb et al 2022b[13]	Yes	Yes	Can't Tell	Yes	Yes	Can't Tell	Can't Tell	Yes	Yes	Yes
Deb et al 2023a[11]	Yes	Yes	Yes	Yes	Yes	Can't Tell	Yes	Yes	Yes	Yes
Deb et al 2023b[12]	Yes	Yes	Can't Tell	Yes	Yes	Can't Tell	Yes	Yes	Yes	Can't Tell
deKuijper et al 2022[14]	Yes	Yes	Can't Tell	No	Yes	Can't Tell	Yes	Yes	Yes	Yes
deWeg et al 2021[15]	Yes	Yes	Can't Tell	Yes	Yes	Can't Tell	Yes	Yes	Yes	Yes

DiBlasi et al 2006[16]	Yes	Yes	Yes	Yes	Yes	Can't Tell	Yes	Yes	Yes	Yes
Doyle. C, 2021[17]	Yes	Yes	Can't Tell	Yes	Can't Tell	Can't Tell	Yes	Yes	Yes	Yes
Doyle. C, 2022,a [18]	Yes	Yes	Yes	Yes	Can't Tell	Can't Tell	Yes	Yes	Yes	Yes
Doyle. C, 2022, b [19]	Yes	Yes	Can't Tell	Yes	Can't Tell	Can't Tell	Yes	Yes	Yes	Yes
Edwards et al 2017[20]	Yes	Yes	Yes	Yes	Can't Tell	Can't Tell	Can't Tell	Yes	Yes	Yes
Erickson et al 2016[21]	Yes	Yes	Yes	Yes	Yes	Can't Tell	Yes	Yes	Yes	Can't Tell
Flood and Henman 202[22]	Yes	Yes	Yes	Yes	Yes	Can't Tell	Can't Tell	Yes	Yes	Yes
Graham et al 2020[23]	Yes	Yes	Yes	Yes	Yes	Can't Tell	Can't Tell	Yes	Yes	Yes
Gupta et al 2021[24]	Yes	Yes	Can't Tell	Can't Tell	Yes	Can't Tell	Can't Tell	Yes	Yes	Yes
Hale et al 2011[25]	Yes	Yes	Can't Tell	Yes	Can't Tell	Can't Tell	Yes	Yes	Yes	Yes
Halmetoja et al 2023[26]	Yes	Yes	Yes	Yes	Yes	Can't Tell	Yes	Yes	Yes	Can't Tell
Heslop et al 2005[27]	Yes	Yes	Can't Tell	Yes	Can't Tell	Can't Tell	Can't Tell	Can't Tell	Yes	Yes
Higgins et al 2015[28]	Yes	Yes	Can't Tell	Yes	Yes	Can't Tell	Yes	Yes	Can't Tell	Yes
Joos et al 2016[29]	Yes	Yes	Can't Tell	Yes	Yes	Can't Tell	Yes	Yes	Yes	Yes
Jothaprasert 2017[30]	Yes	Yes	Can't Tell	Can't Tell	Yes	Can't Tell	Can't Tell	Can't Tell	Yes	Yes
Lake et al 2015[31]	Yes	Yes	Can't Tell	Yes	Yes	Can't Tell	Can't Tell	Can't Tell	Can't Tell	Yes
Lalor and Poulson 2013[32]	Yes	Yes	Yes	Can't Tell	Yes	Can't Tell	Can't Tell	Yes	Yes	Yes
Maine et al 2017[33]	Yes	Yes	Can't Tell	Can't Tell	Yes	Can't Tell	Yes	Yes	Yes	Can't Tell

Marks et al 2021[34]	Yes	Yes	Yes	Yes	Yes	Can't Tell	Yes	Yes	Yes	Yes
Mengoni et al 2016[35]	Yes	Yes	Can't Tell	Yes	Can't Tell	Can't Tell	Yes	Yes	Yes	Yes
Musselwhite-Knell 2008[36]	Yes	Yes	Yes	Yes	Yes	Yes	Yes	Yes	Yes	Yes
Ramerman et al 2018[37]	Yes	Yes	Can't Tell	Yes	Yes	Can't Tell	Can't Tell	Yes	Yes	Can't Tell
Rouse and Finlay 2016[38]	Yes	Yes	Can't Tell	Yes	Yes	Can't Tell	Yes	Yes	Can't Tell	Can't Tell
Sandjojo et al 2019[39]	Yes	Yes	Can't Tell	Yes	Yes	Can't Tell	Yes	Yes	Can't Tell	Can't Tell
Sheehan et al 2019[40]	Yes	Yes	Can't Tell	Yes	Yes	Can't Tell	Yes	Yes	Yes	Yes
Sheehan 2020[41]	Yes	Yes	Can't Tell	Yes	Yes	Can't Tell	Yes	Yes	Yes	Yes
Thompson et al 2013[42]	Yes	Yes	Can't Tell	Yes	Yes	Can't Tell	Yes	Can't Tell	Yes	Yes
Trip et al 2016[43]	Yes	Yes	Yes	Yes	Yes	Can't Tell	Yes	Yes	Yes	Can't Tell
Venables et al 2015[44]	Yes	Yes	Can't Tell	Yes	Can't Tell	Can't Tell	Yes	Can't Tell	Yes	Yes
Whitehead et al 2016[45]	Yes	Yes	Yes	Yes	Yes	Can't Tell	Yes	Yes	Yes	Yes

Q1. Was there a clear statement of the aims of the research?

Q2. Is a qualitative methodology appropriate?

Q3. Was the research design appropriate to address the aims of the research?

Q4. Was the recruitment strategy appropriate to the aims of the research?

Q5. Was the data collected in a way that addressed the research issue?

Q6. Has the relationship between researcher and participants been adequately considered?

Q7. Have ethical issues been taken into consideration?

Q8. Was the data analysis sufficiently rigorous?

Q9. Is there a clear statement of findings?

Q10. How valuable is the research?

Reference for supplementary materials

1. Barratt M, Jorgensen M, Deb S, Limbu B, Donley M, Buchholtz M, et al. Staff perceptions following a training programme about reducing psychotropic medication use in adults with intellectual disability: The need for a realistic professional practice framework. Journal of Applied Research in Intellectual Disabilities 2023:No-Specified. doi: https://dx.doi.org/10.1111/jar.13070
2. Both P, Ten Holt L, Mous S, Patist J, Rietman A, Dieleman G, et al. Tuberous sclerosis complex: Concerns and needs of patients and parents from the transitional period to adulthood. EPILEPSY & BEHAVIOR 2018;83:13-21. doi: 10.1016/j.yebeh.2018.03.012
3. Brown M, Taggart L, Karatzias T, Truesdale M, Walley R, Northway R, et al. Improving diabetes care for people with intellectual disabilities: a qualitative study exploring the perceptions and experiences of professionals in diabetes and intellectual disability services. Journal of Intellectual Disability Research 2017;61(5):435-49. doi: https://dx.doi.org/10.1111/jir.12369
4. Caoili A, Hecker M, Klick S, McLaren J, Beasley J, Barnhill J. Integrated mental health treatment guidelines for prescribers in intellectual and developmental disabilities. Journal of Policy & Practice in Intellectual Disabilities 2023;20(2):164-76. doi: 10.1111/jppi.12447
5. Cardol M, Rijken M, van Schrojenstein Lantman-de Valk H. Attitudes and dilemmas of caregivers supporting people with intellectual disabilities who have diabetes. Patient Education & Counseling 2012;87(3):383-8. doi: https://dx.doi.org/10.1016/j.pec.2011.11.010
6. Cardol M, Rijken M, van Schrojenstein Lantman-de Valk H. People with mild to moderate intellectual disability talking about their diabetes and how they manage. Journal of Intellectual Disability Research 2012;56(4):351-60. doi: https://dx.doi.org/10.1111/j.1365-2788.2011.01472.x
7. Crossley R, Withers P. Antipsychotic medication and people with intellectual disabilities: Their knowledge and experiences. Journal of Applied Research in Intellectual Disabilities 2009;22(1):77-86. doi: https://dx.doi.org/10.1111/j.1468-3148.2008.00454.x
8. Davis SR, Durvasula S, Merhi D, Young PM, Traini D, Bosnic-Anticevich SZ. The role of direct support professionals in asthma management. Journal of Intellectual and Developmental Disability 2015;40(4):342-53. doi: https://dx.doi.org/10.3109/13668250.2015.1041100
9. Davis SR, Durvasula S, Merhi D, Young PM, Traini D, Bosnic Anticevich SZ. Knowledge that people with intellectual disabilities have of their inhaled asthma medications: messages for pharmacists. International Journal of Clinical Pharmacy 2016;38(1):135-43. doi: https://dx.doi.org/10.1007/s11096-015-0217-x
10. Deb S, Limbu B. Support staff liaising effectively with family caregivers: Findings from a co-design event and recommendation for a staff training resource. Frontiers in psychiatry Frontiers Research Foundation 2022;13:977442. doi: https://dx.doi.org/10.3389/fpsyt.2022.977442
11. Deb S, Limbu B, Unwin GL, Weaver T. The use of medication for challenging behaviors in people with intellectual disabilities: The direct care providers' perspective. Journal of Mental Health Research in Intellectual Disabilities 2023:No-Specified. doi: https://dx.doi.org/10.1080/19315864.2023.2192691
12. Deb S, Limbu B, Nancarrow T, Gerrard D, Shankar R. The UK psychiatrists' experience of rationalising antipsychotics in adults with intellectual disabilities: A qualitative data analysis of free-text questionnaire responses. Journal of Applied Research in Intellectual Disabilities 2023;36(3):594-603. doi: 10.1111/jar.13083
13. Deb SS, Limbu B, Unwin GL, Weaver T. Causes of and Alternatives to Medication for Behaviours That Challenge in People with Intellectual Disabilities: Direct Care Providers' Perspectives. International Journal of Environmental Research & Public Health [Electronic Resource] 2022;19(16):13. doi: https://dx.doi.org/10.3390/ijerph19169988
14. de Kuijper G, de Haan J, Deb S, Shankar R. Withdrawing Antipsychotics for Challenging Behaviours in Adults with Intellectual Disabilities: Experiences and Views of Experts by Experience. International Journal of Environmental Research & Public Health [Electronic Resource] 2022;19(23):24. doi: https://dx.doi.org/10.3390/ijerph192315637
15. de Weg JBC, Honingh AK, Teeuw M, Sterkenburg PS. An Exploratory Study among Intellectual Disability Physicians on the Care and Coercion Act and the Use of Psychotropic Drugs for Challenging Behaviour. INTERNATIONAL JOURNAL OF ENVIRONMENTAL RESEARCH AND PUBLIC HEALTH 2021;18(19). doi: 10.3390/ijerph181910240
16. Di Blasi A, Kendall S, Spark MJ. Perspectives on the role of the community pharmacist in the provision of Healthcare to people with intellectual disabilities: Exploration of the barriers and solutions. International Journal of Pharmacy Practice 2006;14(4):263-9. doi: https://dx.doi.org/10.1211/ijpp.14.4.0006
17. Doyle C. 'just knowing' and the challenges of giving medicines to children with severe and profound intellectual disabilities: A hermeneutic inquiry. British Journal of Learning Disabilities 2020:No Pagination Specified. doi: https://dx.doi.org/10.1111/bld.12354
18. Doyle C. The importance of supportive relationships with general practitioners, hospitals and pharmacists for mothers who 'give medicines' to children with severe and profound intellectual disabilities. Journal of

Intellectual Disabilities 2022;26(1):29-49. doi: https://dx.doi.org/10.1177/1744629520951003
19. Doyle. C. Mothers' experiences of giving medicines to children with severe and profound intellectual disabilities—The impact on time. Child: Care, Health & Development 2022;48(4):558-68. doi: 10.1111/cch.12960
20. Edwards N, King J, Watling H, Hair SA. Qualitative exploration of psychotropic medication to manage challenging behaviour in adults with intellectual disability: Views of family members. Advances in Mental Health and Intellectual Disabilities 2017;11(5-6):207-18. doi: https://dx.doi.org/10.1108/AMHID-06-2017-0027
21. Erickson SR, Salgado TM, Tan X. Issues in the Medication Management Process in People Who Have Intellectual and Developmental Disabilities: A Qualitative Study of the Caregivers' Perspective. Intellectual & Developmental Disabilities 2016;54(6):412-26.
22. Flood B, Henman MC. Experiences of the Medication Use Process by People with Intellectual Disabilities. What a Pharmacist Should Know! Pharmacy : A Journal Of Pharmacy Education And Practice 2021;9(1):21. doi: https://dx.doi.org/10.3390/pharmacy9010024
23. Graham YN, Gerrard D, Laight S, Brown R, Keith S, Hayes C. More than medication: Evaluating the role of the pharmacist independent prescriber in a community team for learning disabilities and behaviours deemed to be challenging. British Journal of Learning Disabilities 2020;48(3):232-41. doi: https://dx.doi.org/10.1111/bld.12323
24. Gupta P, McClatchey R, Caleb-Solly P, Ieee. Intelligent IoT System Requirements to Support Self-Management for People with Learning Disabilities - A Study with Care Providers. 2021 17TH INTERNATIONAL CONFERENCE ON INTELLIGENT ENVIRONMENTS (IE). 2021.
25. Hale LA, Trip HT, Whitehead L, Conder J. Self-Management Abilities of Diabetes in People With an Intellectual Disability Living in New Zealand. Journal of Policy & Practice in Intellectual Disabilities 2011;8(4):223-30. doi: 10.1111/j.1741-1130.2011.00314.x
26. Halmetoja A, Teittinen A, Laaksonen R. Challenges and responsibilities in the medication management process in 24/7 group housing services for adults with intellectual disability: Interviews with nurses. Journal of Intellectual Disabilities 2023:17446295231163979. doi: https://dx.doi.org/10.1177/17446295231163979
27. Heslop P, Folkes L, Rodgers J. The knowledge people with learning disabilities and their carers have about psychotropic medication. Learning Disability Review 2005;10(4):10-8. doi: 10.1108/13595474200500033
28. Higgins PS, Shugrue N, Ruiz K, Robison J. Medicare and Medicaid users speak out about their health care: the real, the ideal, and how to get there. Population Health Management 2015;18(2):123-30. doi: https://dx.doi.org/10.1089/pop.2014.0056
29. Joos E, Van Tongelen I, Wijnants K, Mehuys E, Van Bocxlaer J, Remon JP, et al. Drug administration via enteral feeding tube in residential care facilities for individuals with intellectual disability: A focus group study on guideline implementation. Journal of Intellectual Disabilities 2016;20(4):329-40.
30. Jothaprasert R. An analysis of Intellectual Disability [Psy.D. thesis]. Ann Arbor: Alliant International University; 2016.
31. Lake JK, Milovanov A, Sawyer A, Lunsky Y. Parent Perspectives on Psychotropic Medication Use and Interactions with Prescribing Health Care Providers among Adolescents and Adults with an Autism Spectrum Disorder. Focus on Autism and Other Developmental Disabilities 2015;30(3):165-73. doi: https://dx.doi.org/10.1177/1088357614559215
32. Lalor J, Poulson L. Psychotropic medications and adults with intellectual disabilities: Care staff perspectives. Advances in Mental Health and Intellectual Disabilities 2013;7(6):333-45. doi: https://dx.doi.org/10.1108/AMHID-03-2013-0017
33. Maine A, Dickson A, Truesdale M, Brown M. An application of Bandura's 'Four Sources of Self-Efficacy' to the self-management of type 2 diabetes in people with intellectual disability: An inductive and deductive thematic analysis. Research in Developmental Disabilities 2017;70:75-84. doi: https://dx.doi.org/10.1016/j.ridd.2017.09.004
34. Marks AL, Mahoney N, Chen YW, Cordier R, Buchanan A, Wilson NJ. Health promotion challenges for young adults living with intellectual disability and type 1 diabetes. Journal of Intellectual Disabilities 2021:17446295211032767. doi: https://dx.doi.org/10.1177/17446295211032767
35. Mengoni SE, Gates B, Parkes G, Wellsted D, Barton G, Ring H, et al. "Sometimes, it just stops me from doing anything": A qualitative exploration of epilepsy management in people with intellectual disabilities and their carers. Epilepsy & Behavior 2016;64(Pt A):133-9. doi: https://dx.doi.org/10.1016/j.yebeh.2016.09.029
36. Musselwhite-Knell L. Care Workers' Roles in Administering Psychtropic Medication For People With Learning Disabilities in Social Care Homes and Their Views on its Use [D.Clin.Prac. thesis]. Ann Arbor: University of Surrey (United Kingdom); 2008.
37. Ramerman L, Hoekstra PJ, de Kuijper G. Exploring barriers and facilitators in the implementation and use of guideline recommendations on antipsychotic drug prescriptions for people with intellectual disability. Journal of Applied Research in Intellectual Disabilities 2018;31(6):1062-70. doi: https://dx.doi.org/10.1111/jar.12461
38. Rouse L, Finlay WML. Repertoires of responsibility for diabetes management by adults with intellectual disabilities and those who support them. SOCIOLOGY OF HEALTH & ILLNESS 2016;38(8):1243-57. doi: 10.1111/1467-9566.12454

39. Sandjojo J, Gebhardt WA, Zedlitz A, Hoekman J, den Haan JA, Evers AWM. Promoting Independence of People with Intellectual Disabilities: A Focus Group Study Perspectives from People with Intellectual Disabilities, Legal Representatives, and Support Staff. JOURNAL OF POLICY AND PRACTICE IN INTELLECTUAL DISABILITIES 2019;16(1):37-52. doi: 10.1111/jppi.12265
40. Sheehan R, Hassiotis A, Strydom A, Morant N. Experiences of psychotropic medication use and decision-making for adults with intellectual disability: a multistakeholder qualitative study in the UK. BMJ Open 2019;9(11):e032861. doi: https://dx.doi.org/10.1136/bmjopen-2019-032861
41. Sheehan NR. Psychotropic Medication Optimisation in Adults with Intellectual Disability [Ph.D. thesis]. Ann Arbor: University of London, University College London (United Kingdom); 2020.
42. Thompson R, Linehan C, Glynn M, Kerr M. A qualitative study of carers' and professionals' views on the management of people with intellectual disability and epilepsy: a neglected population. Epilepsy Behav 2013;28(379-385.). doi: doi:10.1016/j.yebeh.2013.05.024
43. Trip H, Conder J, Hale L, Whitehead L. The role of key workers in supporting people with intellectual disability in the self-management of their diabetes: a qualitative New Zealand study. Health & Social Care in the Community 2016;24(6):789-98. doi: https://dx.doi.org/10.1111/hsc.12262
44. Venables R, Stirling H, Batchelor H, Marriott J. Problems with oral formulations prescribed to children: a focus group study of healthcare professionals. International Journal of Clinical Pharmacy 2015;37(6):1057-67. doi: https://dx.doi.org/10.1007/s11096-015-0152-x
45. Whitehead LC, Trip HT, Hale LA, Conder J. Negotiated autonomy in diabetes self-management: the experiences of adults with intellectual disability and their support workers. Journal of Intellectual Disability Research 2016;60(4):389-97. doi: https://dx.doi.org/10.1111/jir.12257
