# Supplementary material for: Challenges of using and managing medication: a meta-ethnography of the experiences and perceptions of people with intellectual disability and people who support them
Source: BMJ Open. 2025 Sep 18;15(9):e090876. doi: 10.1136/bmjopen-2024-090876 (PMC12458619; doi:10.1136/bmjopen-2024-090876)
Supplement: Supplementary file 2 [file bmjopen-2024-090876.reviewer_comments.pdf]

PEER REVIEW HISTORY

BMJ Open publishes all reviews undertaken for accepted manuscripts. Reviewers are asked to complete a checklist review form and are provided with free text boxes to elaborate on their assessment. These free text comments are reproduced below.

ARTICLE DETAILS

Title (Provisional)

The challenges of using and managing medication: A meta-ethnography of the experiences and perceptions of people with intellectual disability and people who support them

Authors

Ghosh, Iman; Adams, Danielle; Auguste, Peter; Brown, Anna; Chaplin, Eddie; Flynn, Samantha; Gauly, Julia; Gill, Paramjit; Langdon, Peter; Mahon, David; Martin, Kerry; Patterson, Stephen; Sutherland, Daniel; Tromans, Samuel J; Chen, Yen-Fu; Seers, Kate

---

VERSION 1 - REVIEW

**Reviewer**	**1**
**Name**	**Kelly, Dervla**
**Affiliation**	**University of Limerick, Graduate Entry Medical School**
**Date**	**08-Aug-2024**
**COI**	**n/a**

Thanks you for the invitation to review "The challenges of using and managing medication: A meta- ethnography of the experiences and perceptions of people with intellectual disability and people who support or care for them". While the line of argument synthesis did not yield any new ideas on the topic, the depth of analysis is to be commended. I have made some suggestions to strengthen the paper.

Introduction

Page 5 line 22: I think the reference to diagnostic overshadowing is important. I would encourage the authors to think beyond psychiatric illness and add a reference to reflect this if possible. People with intellectual disability have higher rates of obesity and asthma and lower rates of cancer diagnosed, so it would be useful to broaden the focus.

Suggest the authors add a couple of sentences describing the recent related systematic reviews in the area. Also, has meta ethnography been used to understand medication

management in this population before? It would be nice to describe this to better understand the rationale for the work and highlight the novelty of the approach.

Methods

Eligibility criteria:

Outcomes: Did medication use/management have to be the primary focus of the paper or did the paper just have to have some data on medication use/management?

Phenomenon of interest: Slightly confused by inclusion criteria: I took it from table 1 that papers were included if they were about managing medications in a person with intellectual disability who also has a chronic illness. Then when I look at the included studies, Barrett et al 2023 (REF 54) is about psychotropic management and doesn't mention chronic physical condition. Could the authors clarify this.

Stage 6 & 7: I am struggling to understand how figure 2 and 3 were developed from the methodology. Also, was there evidence from review data of the effectiveness of the proposed solutions in figure 3 or is this hypothetical/derived from the issues idenitfied (e.g. person centred care, shared decision making, effective communication, tools & guidelines…) ? Who contributed to this step?

Results

Page 8, line 39: "The results included a range of participants…" suggest quantify the number of papers that covered each group of participants.

Suggest do a quick analysis and add a description of how many papers focused on management of psychotropic medications and how many focused on managing medications for physical conditions.

Table 2: Where the setting is not specified, did/would you consider following up with authors? Asking as understanding the social setting is such a critical element of ethnographic work.

Figure 1: Notice beside HCPs that nurses and pharmacists are specified. Behavioural therapists and psychologists are increasingly part of health care team. Are these omitted due to data or other reasons? Suggest consider not specifying or removing, more in line with holistic approach.

When attributing quotes "People with intellectual disability" – should this be person with disability?

Page 20: "Monitoring and communication…": I am surprised the challenge of communication between HCPs/not having shared records didn't appear in the results, possibly this section. Was it a code?

Discussion

Page 26: “While the advantages of collaborative work and the need for….”, it would be helpful to understand if the authors believe there is a need for the framework that emphasises collaborative work because they feel this is not happening in practice currently?

Page 26 line 29: Suggest that the authors consider other limitations in the data/analysis, particularity 1) lack of studies/evidence on experiences of people with severe ID with medication; it is difficult to interpret what a ‘good’ experience of medication looks like for this cohort, particularity with qualitative interview studies; 2) further exploration on the impact of setting on medication use and management would be invaluable e.g. community vs residential. See later comment also.; 3) data is very focused on psychiatric medications. I’m not sure how robust this is to generalise to other conditions e.g. asthma, obesity, rheumatoid arthritis, cancer. Suggest some reflection and comment on this.

Page 26, line 35. There is new material about methods introduced in paragraph 4 on page 26 which needs to also be included in the methods… coherence and adequacy information.

Overall I think the conceptual framework goes at least some of the way to representing the complexity of why people take certain medications. I also agree that the quality of the relationships between actors in the ‘collective’ is integral to successful use of medications, and think this point cannot be emphasised enough. Making decisions about medication (and understanding manifestation of illness) is also shaped by personal histories of patients e.g. experiences of poverty and trauma. We are hoping to publish a paper with some evidence of this in the context of ID. I personally think that successful personalised care is difficult to achieve without addressing some of these factors, and it can be difficult to tease out the impact of these on presentation for treatment. It may be something to consider for the discussion / future work.

**Reviewer**	**2**
**Name**	**Erickson, Steven R.**
**Affiliation**	**Univ Michigan**
**Date**	**11-Aug-2024**
**COI**	**None**

Overall impression: I highly commend the investigators for their work on this extremely important issue. They have done an excellent job of examining the literature around medication management issues for persons with intellectual or developmental disabilities, using acceptable and rigorous approach, to create a framework that is easy to view and interpret. I am very impressed with the project’s scope and outcomes.

I honestly have very little to comment on for this manuscript. The introduction is written in a way to guide the reader through the issues and why this project was undertaken. The methods are well described and referenced. The results appear transparent, thorough, and

are easy to follow. Highlighting themes with examples from qualitative assessments is very useful to make the point.

The discussion summarizes the findings and provides guideance on how to use the information.

One specific comment has to do with the inclusion of experts by experience. There is mention on pages 5/6 lines 57 through 5 (next page) about persons with IDD being involved. There is a very brief description of their involvement. First, it is vital that all research on persons with disabilities should be informed by those with disabilities, which the investigative team did. In studies that do have co investigators or informant/advisory members with disabilities, this strength should be called out in the discussion section. Also, in either results or discussion, there may be some discussion of how the members of the group actually impacted the process.

At the end of the manuscript, it seems to end with the limitations section, rather than a conclusion section. The conclusion found in the abstract is excellent; could it or a revised wording be used for the manuscript itself? Help out the clinicians reading the paper on how to use this fine and important information.

**Reviewer**	**3**
**Name**	**Nguyen, Linda**
**Affiliation**	**McMaster University**
**Date**	**28-Sep-2024**
**COI**	**I have no conflicts of interest to declare.**

Thank you to the authors for conducting this important qualitative review about the use and management of medications. The review was very comprehensive in providing an understanding about this topic using a meta-ethnography approach.

I provided some feedback to further strengthen this paper:

- In the Introduction, the authors mentioned about the multifaceted issues related to managing medications. Have there been other reviews conducted about this topic? How might understanding the perspectives of people with intellectual disability, their family and paid carers, and health and social professionals complement and build on the existing literature (e.g., other studies and reviews)?

- I appreciate that the authors consulted with a Project Advisory Group. How was this group formed? Or was this group an existing group? What were some examples of ways that the Project Advisory Group contributed to the steps of the meta-ethnography? I am curious about how the dynamics of the group were navigated. For example, how was an

environment created where the adult with an intellectual disability would feel comfortable to provide equal and valuable input as the other health and social professionals?

- The authors described the Project Advisory Group, as well as two Patient and Public Involvement (PPI) groups. Could the authors clarify the differences between the groups? How did the authors decide on having two PPI groups? Were there any disagreements between the perspectives of the researchers and PPI groups? If so, how were these disagreements addressed?

- The authors synthesized the results from the perspectives of paid and unpaid carers, including family members. Were the family members primarily parents? Or were there other family member roles (e.g., siblings)?

- For Stage 5 - Translating studies into each other, were there new themes that the Project Advisory Group raised that were not initially identified? How were the themes refined based on input from the Project Advisory Group?

- I appreciate how the authors presented an initial conceptual framework with Figure 1. Could the authors describe the process for how Figure 2 was derived from Figure 1? A specific question to Figure 1 is the concept of tension, could the authors clarify who the tension was with and where the tension stemmed from? How did Figure 1 inform the process for understanding and synthesizing the results of the included studies? How was Figure 2 created?

- Figure 3 provides a conceptual model to illustrate the overarching concept of 'collective collaboration'. Is Figure 3 presented as a way to complement Figure 2?

- The authors included studies with children, adolescents and adults with intellectual disability, as well as their carers and health and social care professionals who support them. Were there differences in the types of medication support required based on the age of the individual with an intellectual disability? For example, did age have a role in the theme of navigating autonomy and relationships?

- Theme 3 highlighted the barriers to knowledge and training needs for medication among people with intellectual disability and their carers, while Theme 4 highlighted the lack of accessible and tailored information in healthcare systems. Did the included studies highlight the differences in the transition between healthcare systems, such as the transition from pediatric to adult healthcare and the training needs that youth with intellectual disabilities and their carers may require to prepare for the changes in access to medications?

- The overarching concept of 'collective collaboration' was highlighted with the need to improve communication and positive relationships in the healthcare system to support individuals with intellectual disabilities in the management of medications. It may be helpful for the authors to highlight key recommendations in the Discussion section for how to address 'collective collaboration', for example, suggestions for healthcare professionals to address this concept in their practice.

- There is a minor grammatical correction for Table 2: Page 14, line 14: “Care” for the sentence, “care professionals (three project managers, two project co-ordinators, and two support workers” should be capitalized to be consistent with the formatting of the table.

**Reviewer**	**4**
**Name**	**Doyle, carmel**
**Affiliation**	**TCD**
**Date**	**31-Mar-2025**
**COI**	**No competing interests.**

This is an interesting article and demonstrates a well-executed meta ethnography. You detail the formulaic process for this well. The following comments are aimed at developing this piece of work further:

• Title – do you need the phrase ‘or care for’. Is support them not enough?

• Findings while all relevant are somewhat heavily laden with quotes presented together in text. Can you use an intro line to split quotes?

• Your conceptual framework gives a graphically sound summary of the key issues and I like the way this is developed and reflective of findings.

• Your discussion is ‘light’ and a reconsideration of more depth would be helpful for this section.

VERSION 1 - AUTHOR RESPONSE

Reviewer: 1
Dr. Dervla Kelly, University of Limerick
Comments to the Author:

#4
Thanks you for the invitation to review “The challenges of using and managing medication: A meta- ethnography of the experiences and perceptions of people with intellectual disability and people who support or care for them”. While the line of argument synthesis did not yield any new ideas on the topic, the depth of analysis is to be commended. I have made some suggestions to strengthen the paper.

Author’s response: Thank you very much for your comments and constructive suggestions, which are very helpful for us to further strengthen the manuscript.

Introduction
#5
Page 5 line 22: I think the reference to diagnostic overshadowing is important. I would

encourage the authors to think beyond psychiatric illness and add a reference to reflect this if possible. People with intellectual disability have higher rates of obesity and asthma and lower rates of cancer diagnosed, so it would be useful to broaden the focus.

Author's response: Thank you for the comment. We have now highlighted that diagnostic overshadowing covers both physical and mental conditions and have added references related to cancer and cardiovascular conditions in 'Introduction'; page 3.

#6
Suggest the authors add a couple of sentences describing the recent related systematic reviews in the area. Also, has meta ethnography been used to understand medication management in this population before? It would be nice to describe this to better understand the rationale for the work and highlight the novelty of the approach.

Author's response: Thank you for your feedback. we conducted an additional search to identify relevant systematic reviews and meta-ethnographies. While there are some related reviews, which we have cited, none made use of meta-ethnography; this makes our work unique and we have highlighted our use of meta-ethnography in the 'Introduction' and in the 'Strengths and limitations of this study' sections.

Methods

#7
Eligibility criteria:
Outcomes: Did medication use/management have to be the primary focus of the paper or did the paper just have to have some data on medication use/management?

Author's response: Thank you. Our primary focus was on data relating to the perceptions and experiences of medication use and management. These did not have to be the central focus of the included studies. We have now added the following detail to Table 1: "Studies reporting relevant qualitative on these outcomes were considered, whether or not the data were the central focus of the studies (e.g. studies focusing on disease management for which medication management is an important part would also be included)."

#8
Phenomenon of interest: Slightly confused by inclusion criteria: I took it from table 1 that papers were included if they were about managing medications in a person with intellectual disability who also has a chronic illness. Then when I look at the included studies, Barrett et al 2023 (REF 54) is about psychotropic management and doesn't mention chronic physical condition. Could the authors clarify this.

Author's response: Thank you for your comment. We have now made it clearer in Table 1 that either those with a long-term physical condition or those with chronic mental health issues and/or challenging behaviours for which medication was prescribed were of interest for this meta-ethnography.

#9

Stage 6 & 7: I am struggling to understand how figure 2 and 3 were developed from the methodology. Also, was there evidence from review data of the effectiveness of the proposed solutions in figure 3 or is this hypothetical/derived from the issues idenitfied (e.g. person centred care, shared decision making, effective communication, tools & guidelines...) ? Who contributed to this step?

Author's response: Thank you. Our first conceptual model shown in Figure 1 provides an overview of main conceptual categories identified from the data and illustrates how they are related to each other in terms of different people involved in the medication use process, main issues concerned with each group of people as well as issues arising from the interactions between them. However, in order to move beyond the descriptive account of the conceptual categories emerged from the data, we tried to simplify and distil these categories into four higher-level, cross-cutting thematic groups. In the process of further exploration of the connectedness of the conceptual categories and thematic groups, we identified the three key aspects of medication use (context, person and medication) that are related to and reflected in all the thematic groups, and incorporated these with the thematic groups to form our second conceptual model. We then combined the findings from the first two conceptual models with understanding of the medication use process that we gained from the literature as well as experiences shared by our PPI and Project Advisory groups and translated them into our line of argument (of what might impede optimal medication use among people with intellectual disabilities and how might these be overcome) and the overarching concept of 'collective collaboration'., which was presented in the final model in Figure 3. You have correctly flagged up that the proposed solutions were based on our findings from qualitative evidence and therefore may require testing of their effectiveness to generate empirical evidence. We have now explained these in relevant 'Methods' (stage 6 and 7; page 6) and 'Results' (Overarching concept; page 14-15) sections and have identified the contributing reviewers for this part of synthesis.

Results

#10

Page 8, line 39: "The results included a range of participants..." suggest quantify the number of papers that covered each group of participants.

Author's response: Thank you. We have now quantified the number of studies contributing to each category of participants.

#11

Suggest do a quick analysis and add a description of how many papers focused on management of psychotropic medications and how many focused on managing medications for physical conditions.

Author's response: Thank you for your comment. We had already provided a brief description and referenced the corresponding studies in the 'Result' section (Page 7). As suggested, we have now included the number of studies in each category for greater clarity.

#12
Table 2: Where the setting is not specified, did/would you consider following up with authors? Asking as understanding the social setting is such a critical element of ethnographic work.

Author's response: Thank you very much. Since submitting this work, we have revisited the 'setting' and recoded some studies with more details to better describe the context of the reported research. We have now included information concerning participant recruitment as reported by the authors and reported the settings that we inferred from such information for studies previously recorded as 'Not specified' for settings. Accordingly, we have now updated the study characteristics table in *supplementary material; table 1*. We had not needed to contact authors for further information.

#13
Figure 1: Notice beside HCPs that nurses and pharmacists are specified. Behavioural therapists and psychologists are increasingly part of health care team. Are these omitted due to data or other reasons? Suggest consider not specifying or removing, more in line with holistic approach.

Author's response: Thank you for highlighting this point. Indeed, behavioural therapists and psychologists were not included in our conceptual model in Figure 1 because they were not featured in the qualitative data that we reviewed. Nevertheless, we concur that inputs from additional staff of a multidisciplinary team including behavioural therapists and psychologists could be very important. We prefer keeping Figure 1 as it is to reflect evidence from included studies, but have added the following text to the Discussion section: "In terms of integrative care incorporating 'collective collaboration' that is described in our conceptual models, multidisciplinary involvement (in addition to prescribers, pharmacists and nurses) such as inputs from behavioural therapists, psychologists, and members of staff from education institutions and social services is increasingly common and could be very important to ensure a holistic approach. These may not have been sufficiently featured in our conceptual models and should be further integrated into future revisions and refinement of the models as required.

#14
When attributing quotes "People with intellectual disability" – should this be person with disability?

Author's response: Thank you for flagging this up. We have corrected the error for relevant quotes in the manuscript.

#15
Page 20: "Monitoring and communication…": I am surprised the challenge of communication between HCPs/not having shared records didn't appear in the results, possibly this section. Was it a code?

Author's response: Thank you for your comment. We found evidence of gaps in communication between HCPs and carers, particularly in group home settings, where client records (e.g., medical appointments, medication indications) were not consistently shared. We clearly presented this issue in the conceptual model in Figure 1. Issues related to communication of information were also explicitly or implicitly represented in various parts of Figure 2, with

effective communication highlighted as an important element of collective collaboration in Figure 3. We did not come across data specifically related to communication issues between HCPs but were aware of the issue of lack of shared records between HCPs (e.g. between prescribers and pharmacists conducting medication reviews) as you highlighted in a separate evidence synthesis of quantitative feasibility studies of medication support interventions. We have now added the following text to acknowledge the potential limitation: "As the research question that we were trying to address was very broad, we might not have covered and described all specific issues in the medication use process for people with intellectual disabilities, for example issues related to communication of information between different groups of people involved or issues associated with transition between different services and care settings. Nevertheless, we hope the overarching concept and key themes and aspects of care described in our line of argument and related conceptual models are sufficiently comprehensive to be applicable across diverse scenarios."

Discussion

#16

Page 26: "While the advantages of collaborative work and the need for….", it would be helpful to understand if the authors believe there is a need for the framework that emphasises collaborative work because they feel this is not happening in practice currently?

Author's response: Thank you, yes this is the case. We have now described this more clearly in the second paragraph of the 'Discussion' section; page 15-16.

#17

Page 26 line 29: Suggest that the authors consider other limitations in the data/analysis, particularity 1) lack of studies/evidence on experiences of people with severe ID with medication; it is difficult to interpret what a 'good' experience of medication looks like for this cohort, particularity with qualitative interview studies; 2) further exploration on the impact of setting on medication use and management would be invaluable e.g. community vs residential. See later comment also.; 3) data is very focused on psychiatric medications. I'm not sure how robust this is to generalise to other conditions e.g. asthma, obesity, rheumatoid arthritis, cancer. Suggest some reflection and comment on this.

Author's response: Thank you very much for your comment. We have incorporated your helpful suggestions and expand on these issues in various parts of the 'Discussion' section.

#18

Page 26, line 35. There is new material about methods introduced in paragraph 4 on page 26 which needs to also be included in the methods… coherence and adequacy information.

Author's response: Thank you. We have now introduced the terms 'coherence' and 'adequacy' in the 'Methods' section; page 5, where we describe how we operationalised the GRADE-CERQual approach in more detail.

#19
Overall I think the conceptual framework goes at least some of the way to representing the complexity of why people take certain medications. I also agree that the quality of the relationships between actors in the 'collective' is integral to successful use of medications and think this point cannot be emphasised enough. Making decisions about medication (and understanding manifestation of illness) is also shaped by personal histories of patients e.g. experiences of poverty and trauma. We are hoping to publish a paper with some evidence of this in the context of ID. I personally think that successful personalised care is difficult to achieve without addressing some of these factors, and it can be difficult to tease out the impact of these on presentation for treatment. It may be something to consider for the discussion / future work.

Author's response: Thank you for sharing this insight. We look forward to reading your upcoming paper on the topic. We have now incorporated this point into the description of the overarching concept: "Understanding a person's underlying condition(s) and/or behaviour(s) for which medication is being considered or prescribed, along with other health needs and preferences, is the starting point for medication use. These could include a person's past history related to illnesses, treatments and other life events and circumstances such as poverty and trauma that may have influenced manifestation of disease conditions, behaviours and medication related decisions".

Reviewer: 2
Dr. Steven R. Erickson, Univ Michigan
Comments to the Author:

#20
Overall impression: I highly commend the investigators for their work on this extremely important issue. They have done an excellent job of examining the literature around medication management issues for persons with intellectual or developmental disabilities, using acceptable and rigorous approach, to create a framework that is easy to view and interpret. I am very impressed with the project's scope and outcomes.
I honestly have very little to comment on for this manuscript. The introduction is written in a way to guide the reader through the issues and why this project was undertaken. The methods are well described and referenced. The results appear transparent, thorough, and are easy to follow. Highlighting themes with examples from qualitative assessments is very useful to make the point. The discussion summarizes the findings and provides guidance on how to use the information.

Author's response: Thank you for your comments recognising the value of this piece of work.

#21
One specific comment has to do with the inclusion of experts by experience. There is mention on pages 5/6 lines 57 through 5 (next page) about persons with IDD being involved. There is a very brief description of their involvement. First, it is vital that all research on persons with disabilities should be informed by those with disabilities, which the investigative team did. In studies that do have co investigators or informant/advisory members with disabilities, this strength should be called out in the discussion section. Also, in either results or discussion, there may be some discussion of how the members of the group actually impacted the process. At the end of the manuscript, it seems to end with the limitations section, rather than a

conclusion section. The conclusion found in the abstract is excellent; could it or a revised wording be used for the manuscript itself?   Help out the clinicians reading the paper on how to use this fine and important information.

Author's response: Thank you very much for your comments and suggestions. This meta-ethnography forms part of a broader evidence synthesis project that has incorporated the insights and contributions of experts by experience at various stages throughout. To highlight the impact of their inputs and to ensure transparency in presenting our analysis, we have now included additional descriptions of the involvement and influence of the PPI and project advisory groups within the Results and highlighted as a key strength in the Discussion sections. For example, we have highlighted that the understanding of medication use process that we gained from the experience shared by our PPI groups has helped shape our line of argument and the overarching concept of 'collective collaboration' alongside the evidence from the literature.

A conclusion is also added as suggested in page 17.

Reviewer: 3
Ms. Linda Nguyen, McMaster University
Comments to the Author:

#22
Thank you to the authors for conducting this important qualitative review about the use and management of medications. The review was very comprehensive in providing an understanding about this topic using a meta-ethnography approach.
Author's response: Thank you very much for your comments.

#23
I provided some feedback to further strengthen this paper:
- In the Introduction, the authors mentioned about the multifaceted issues related to managing medications. Have there been other reviews conducted about this topic? How might understanding the perspectives of people with intellectual disability, their family and paid carers, and health and social professionals complement and build on the existing literature (e.g., other studies and reviews)?

Author's response: Thank you. Following your and Reviewer 1's feedback, we conducted an additional search to identify relevant systematic reviews and meta-ethnographies. However, we did not find any reviews or meta-ethnographies that addressed the topic with a broader scope. We have now briefly describe other relevant reviews (as mentioned in our response to comment #6 of Reviewer 1 above), and have more clearly described the motivation and strength for our approach: "Through examination of qualitative evidence from different groups of people involved in the use and management of medications for people with intellectual disabilities across different conditions and indications using meta-ethnography, this review aimed to develop an in-depth and more holistic understanding of potential challenges in optimising medication use among people with intellectual disabilities and to suggest ways to overcome the challenges, which could provide new insights that might not have been uncovered in previous reviews adopting narrower scopes and narrative synthesis approaches.

#24
- I appreciate that the authors consulted with a Project Advisory Group. How was this group formed? Or was this group an existing group? What were some examples of ways that the Project Advisory Group contributed to the steps of the meta-ethnography? I am curious about how the dynamics of the group were navigated. For example, how was an environment created where the adult with an intellectual disability would feel comfortable to provide equal and valuable input as the other health and social professionals?

Author's response: Thank you for your comment. The SMILE project, funded by the National Institute for Health and Care Research in the United Kingdom , aims to provide a comprehensive evaluation and summary of evidence surrounding medication support strategies for people with intellectual disabilities and their carers. The project comprises multiple components, including this meta-ethnography and a scoping review, a systematic review of intervention effectiveness and another systematic review on intervention feasibility and implementation.

Our Project Advisory Group was established specifically as part of the planned project, consisting of people with intellectual disabilities, staff members from charitable organisations and healthcare professionals with expertise in supporting people with intellectual disabilities, including a clinical psychologist, a psychiatrist, a general practitioner, a pharmacist and a nurse; many of the Project Advisory Group members were also senior academics with research expertise in this subject area. The chair of the Advisory Group and other group members were all familiar with communicating with members of experts by lived experience, making appropriate adjustments such as avoiding jargons and actively inviting their contribution in the group meetings. This group was involved from the outset, contributing to the development of the grant application and providing ongoing oversight of the project. The Project Advisory Group reviewed and discussed emerging themes and early version of conceptual models developed by the research review team, and provided comments that directly informed the refinement of the conceptual models.

In addition, two patient and public involvement (PPI) groups were formed as part of the broader project. One group included people with intellectual disabilities. Meetings of the PPI group was facilitated by the project's PPI lead, who had substantial experience in working with people with intellectual disabilities as a project manager in relevant charity. Meetings were conducted virtually, allowing participants to remain in familiar environments. Participants with intellectual disabilities were supported by workers who have been supporting them outside the research project during (or sometimes before/after) the meeting. Regular breaks were built into the sessions to accommodate participants' needs and ensure their comfort and engagement. The second PPI group consisted of family carers and health and social care professionals who support people with intellectual disability, with separate meetings conducted to gather their perspectives. The project PPI lead was responsible for facilitating communication, coordinating meetings, and supporting the participation of both PPI groups throughout the project.

We have now included the above detailed description of the contributions of the PPI groups and the Project Advisory Group towards this meta-ethnography in the *supplementary material 2*. Additionally, a brief summary of their contributions has now been included in the Results section as described in our earlier responses to comments by other reviewers.

#25

- The authors described the Project Advisory Group, as well as two Patient and Public Involvement (PPI) groups. Could the authors clarify the differences between the groups? How did the authors decide on having two PPI groups? Were there any disagreements between the perspectives of the researchers and PPI groups? If so, how were these disagreements addressed?

Author's response: Thank you. As described above, the Project Advisory Group comprised of individuals representing key stakeholders with extensive experience working with people with learning disabilities and their carers, with a strong focus on research in this field and relevant methodological expertise. This group played a vital role in shaping the research questions, refining the methodology, and offering expert advice and guidance throughout the broader project.

By contrast, the two PPI groups made contribution towards the meta-ethnography and the border project mainly by sharing their lived experiences, helping the research review team to become sensitised to issues of importance to them (as service users) and to contextualise and relate the research findings to real-world practice. In addition they helped identifying issues important for them that did not seem to have been adequately covered by research literature that we identified. The two PPI groups were pre-planned, integral parts of the project. Three charitable organisations with established experience and expertise in supporting and/or advocating for people with intellectual disabilities were contracted as collaborators of the research project to facilitate recruitment of PPI group members and to provide ongoing support for their participation in the PPI group activities. We deliberated created two groups, separating people with intellectual disabilities from their carers and health/care professionals to ensure that they were able to freely share their views and experience without being inhibited by the presence of the latter members. Possibly because we were working with rather than doing research on PPI group members, the research review team found inputs from the PPI groups very helpful in enhancing research reviewers' understanding of evidence reported in the literature and in contextualise and validating the findings. We did not encounter disagreements in perspectives between the PPI groups and the researchers. A few suggestions made by the PPI groups fell outside the scope of our project, but we were able to incorporate them as recommendations for further research for the wider project. Findings of the meta-ethnography were shared with the PPI groups as part of our efforts to acknowledge and honour their valuable contributions to the research, and PPI group members were generally in agreement that the findings reflected their lived experience and issues that they cared about. We have now included the above information in *supplementary material 2*.

#26

- The authors synthesized the results from the perspectives of paid and unpaid carers, including family members. Were the family members primarily parents? Or were there other family member roles (e.g., siblings)?

Author's response: Thank you for your comment. We have presented available information regarding study participants in the study characteristic table in *supplementary material; table 1*. Not all studies provided sufficient description about family carers; where reported, the family members were indeed primarily parents (and mostly, mothers) as you rightly suggested. Sibling

carers were included in a few studies but no unique issues or experiences from their perspective were reported in the included studies.

#27
- For Stage 5 - Translating studies into each other, were there new themes that the Project Advisory Group raised that were not initially identified? How were the themes refined based on input from the Project Advisory Group?

Author's response: Thank you. This meta-ethnography synthesises findings from published literature, and the overarching concepts were developed based on the data reported in the included studies. The Project Advisory Group and the PPI groups were involved in validating the findings including identified themes and preliminary conceptual models by reflecting on them in light of their lived experiences. As their role was focused on validation rather than data generation, no additional themes were identified through the Advisory and PPI Group input. However, some refinement in wording and configuration of the conceptual models were informed by their comments. We have now provided a brief description of their contribution in the Results section; page 14, with further details included in the *supplementary material 2*.

#28
- I appreciate how the authors presented an initial conceptual framework with Figure 1. Could the authors describe the process for how Figure 2 was derived from Figure 1? A specific question to Figure 1 is the concept of tension, could the authors clarify who the tension was with and where the tension stemmed from? How did Figure 1 inform the process for understanding and synthesizing the results of the included studies? How was Figure 2 created?

Author's response:- Thank you for your comment. As described in our response to comment #9 from Reviewer 1, Figure 1 summarised descriptive conceptual categories emerged from the qualitative data and their relationships from the perspectives of different actors involved in the medication use process, and Figure 2 was created as a result of our attempt to distil and simplify the conceptual categories to generate higher-level thematic groups and to identify key aspects of medication use that cut across these conceptual categories and thematic groups. We have now provided more detailed description regarding the development of the conceptual models in the 'Method' *(*stage 6 and 7; page 6) section as well as in the 'Results' (overarching concept; page 14-15) section.

In Figure 1, the concept of 'tension' was mainly derived from the potential conflict between the preference of people with intellectual disabilities and the actions taken by their caregivers based on their judgement of the best interest for the people whom they care for in relation to disease management and medication use. Data were mostly from the direct carer (family members), as illustrated by the description of having to act as "lifestyle police" by caregivers. Inner tension could also be experienced by caregivers when they needed to take responsibility for making a decision that requires balancing between potential risk and benefit of medication use and/or between the autonomy of people with intellectual disabilities and their best interest as judged by the caregivers.

#29
- Figure 3 provides a conceptual model to illustrate the overarching concept of 'collective collaboration'. Is Figure 3 presented as a way to complement Figure 2?

Author's response: As explained in our response to comment #9 of Reviewer 1 above, Figure 3 was built upon the two conceptual models derived from qualitative data that we reviewed and our understanding of the medication use process obtained from the literature and the experiences shared by our PPI and Advisory groups. It was created to translate our findings into a line of argument with regard to what might hinder optimal use of medication and what solutions might overcome these barriers, and to illustrate the overarching concept of 'collective collaboration' that sums up the key essence.

#30

- The authors included studies with children, adolescents and adults with intellectual disability, as well as their carers and health and social care professionals who support them. Were there differences in the types of medication support required based on the age of the individual with an intellectual disability? For example, did age have a role in the theme of navigating autonomy and relationships?

Author's response: Thank you for highlighting this important point. The eligible articles included in our meta-ethnography provided fairly limited information on the level of support according to age groups. Therefore, we were unable to further explore the impact of age groups on medication use issues and collaborative practices. As described in our response to comment #15 from Reviewer 1, we have now acknowledged as a limitation of our review that we might not have captured and described all specific issues related to medication use in different patient groups and settings, but we hope that our conceptual models and line of argument may be comprehensive enough to be applicable across diverse scenarios. We have also suggested that "Further work to explore and explicate how different contexts (e.g., community vs residential settings; different age groups and people with different severity of intellectual disabilities) impact upon the manifestation of different issues and the needs for and practicality of different solutions would be invaluable."

#31

- Theme 3 highlighted the barriers to knowledge and training needs for medication among people with intellectual disability and their carers, while Theme 4 highlighted the lack of accessible and tailored information in healthcare systems. Did the included studies highlight the differences in the transition between healthcare systems, such as the transition from paediatric to adult healthcare and the training needs that youth with intellectual disabilities and their carers may require to prepare for the changes in access to medications?

Author's response: Thank you for your valuable comments. We analysed and developed themes based on the data (quotes) reported in the included studies. There was substantial data highlighting training and education needs, while data related to healthcare accessibility was relatively limited. We did not identify any relevant perspectives on the transition from paediatric to adult care within included studies.

As part of the wider project, we conducted a systematic review of feasibility evaluation of various interventions and identified issues specifically related to the transition between paediatric and adult healthcare. However, no data were reported that addressed training needs during this transitional period. We have now included lack of data on issues associated with

transition between services and care settings as a potential limitation of this work, for which further research would be very valuable.

#32
- The overarching concept of 'collective collaboration' was highlighted with the need to improve communication and positive relationships in the healthcare system to support individuals with intellectual disabilities in the management of medications. It may be helpful for the authors to highlight key recommendations in the Discussion section for how to address 'collective collaboration', for example, suggestions for healthcare professionals to address this concept in their practice.

Author's response: Thank you for your comment. In line with the higher-level synthesis of evidence through our meta-ethnography, we have listed various principles and approaches to addressing collective collaboration, such as shared decision making and effective communication, in the conceptual model presented in Figure 3. We hope that practitioners and policy makers can adopt these principles and choose from these approaches to develop strategies that will suit their individual contexts. A recommendation for applying the conceptual framework and related principles has now been included at the end of the Discussion section.

#33
- There is a minor grammatical correction for Table 2: Page 14, line 14: "Care" for the sentence, "care professionals (three project managers, two project co-ordinators, and two support workers" should be capitalized to be consistent with the formatting of the table.

Author's response: Thank you. We have now corrected the typo.

Reviewer: 4
Dr. carmel Doyle, TCD
Comments to the Author:

#34
This is an interesting article and demonstrates a well-executed meta ethnography. You detail the formulaic process for this well. The following comments are aimed at developing this piece of work further:

Author's response: Thank you very much for your comments and suggestions.

#35
• Title – do you need the phrase 'or care for'. Is support them not enough?

Author's response: Thank you for your comment. We agreed that the phrase was not essential and have revised our title to make it more concise.

#36
• Findings while all relevant are somewhat heavily laden with quotes presented together in text. Can you use an intro line to split quotes?

Author's response: Thank you for this helpful suggestion. We have now reformatted the text and added an intro line to separate main texts from the quotes for better readability.

#37
• Your conceptual framework gives a graphically sound summary of the key issues and I like the way this is developed and reflective of findings.

Author's response: Thank you for your kind appreciation of our work.

#38
• Your discussion is 'light' and a reconsideration of more depth would be helpful for this section.

Author's response: Thank you. We have substantially expanded the Discussion section in response to your comment and comments from the other reviewers to provide a more in-depth discussion on the strengths and limitations of the study, and to highlight additional issues that may be worth considering. We have also outlined the implications of our findings for practice, policy, and future research more clearly.
